# Genome Characterization and Phylogenetic Analysis of Scale Drop Disease Virus Isolated from Asian Seabass (*Lates calcarifer*)

**DOI:** 10.3390/ani14142097

**Published:** 2024-07-18

**Authors:** Putita Chokmangmeepisarn, Mohammad Noor Amal Azmai, Jose A. Domingos, Ronny van Aerle, David Bass, Pochara Prukbenjakul, Saengchan Senapin, Channarong Rodkhum

**Affiliations:** 1 Center of Excellence in Fish Infectious Diseases (CE FID), Faculty of Veterinary Science, Chulalongkorn University, Bangkok 10330, Thailand; 2 Department of Biology, Faculty of Science, Universiti Putra Malaysia, UPM, Serdang 43400, Selangor, Malaysia; mnamal@upm.edu.my; 3 Aquatic Animal Health and Therapeutics Laboratory, Institute of Bioscience, Universiti Putra Malaysia, UPM, Serdang 43400, Selangor, Malaysia; 4 Tropical Futures Institute, James Cook University, Singapore 387370, Singapore; jose.domingos1@jcu.edu.au; 5 International Centre of Excellence for Aquatic Animal Health, Centre for Environment, Fisheries and Aquaculture Sciences (Cefas), Weymouth, Dorset DT4 8UB, UK; ronny.vanaerle@cefas.gov.uk (R.v.A.); david.bass@cefas.gov.uk (D.B.); 6 Centre for Sustainable Aquaculture Futures, University of Exeter, Stocker Road, Exeter EX4 4QY, UK; 7 Department of Life Sciences, The Natural History Museum, London SW7 5BD, UK; 8 Fish Health Platform, Center of Excellence for Shrimp Molecular Biology and Biotechnology (Centex Shrimp), Faculty of Science, Mahidol University, Bangkok 10400, Thailand; 9 National Center for Genetic Engineering and Biotechnology (BIOTEC), National Science and Technology Development Agency (NSTDA), Khlong Nueng, Pathum Thani 12120, Thailand

**Keywords:** scale loss, metagenomics, evolution, mutations

## Abstract

**Simple Summary:**

Scale drop disease virus (SDDV) has been identified as a significant pathogen causing scale drop syndrome (SDS), leading to significant economic losses in Asian seabass production in Southeast Asia. This study utilized a metagenomic approach to investigate the bacterial and viral communities associated with SDS, with a particular focus on SDDV, and evaluated the potential of metagenomics for retrieving complete SDDV genomes. By characterizing the complete genomes of SDDV strains, we aimed to gain a deeper understanding of the virus. The insights gained from this study are expected to inform the development of comprehensive disease prevention and control strategies for SDDV, mitigating its impact on the aquaculture industry.

**Abstract:**

Scale drop disease virus (SDDV), a double-stranded DNA virus in the family *Iridoviridae*, has been reported widely in southeast Asian countries as a causative agent of scale drop syndrome (SDS) in Asian seabass. SDS has resulted in high mortality and significant economic losses to the aquaculture industry. This study demonstrated the use of metagenomic methods to investigate bacterial and viral communities present in infected fish tissues and recover a complete genome of the causative agent named SDDV TH7_2019. Characterization of the TH7_2019 genome revealed a genome size of 131 kb with 134 putative ORFs encoding viral proteins potentially associated with host apoptosis manipulation. A comparative genome analysis showed a high degree of amino acid identity across SDDV strains, with variations in number of repeat sequences and mutations within core genes. Phylogenetic analyses indicate a close relationship among SDDV genomes. This research enhances our understanding of the genetic diversity and evolutionary relationship of SDDV, contributing valuable insights for further development of effective control strategies of SDDV.

## 1. Introduction

Asian seabass (*Lates calcarifer*) is recognized as a euryhaline species, thriving in both brackish and nearshore marine environments. This species has considerable economic importance within the Asia-Pacific region. It is widely cultured in Australia, Singapore, Malaysia, Thailand, Indonesia, and China [1]. However, intensive aquaculture practices have resulted in a notable impact on Asian seabass cultures, particularly in their susceptibility to infectious diseases. Scale drop syndrome (SDS) represents a significant threat to aquaculture, particularly for Asian seabass cultivation. This disease was initially documented in an Asian seabass farm in Malaysia in 1992 [2]. Typically, affected fish exhibit scale loss over extensive areas, accompanied by skin discoloration, darkened bodies, gill pallor, tail and fin erosion, as well as pathological features such as vasculitis and tissue necrosis in major internal organs [3]. Several pathogens have been implicated in causing SDS in Asian seabass, including scale drop disease virus (SDDV), *Vibrio harveyi*, and *Tenacibaculum maritimum* [2,4,5,6,7,8]. However, the current scientific consensus is that SDS in Asian seabass is caused by SDDV, resulting in significant economic losses in production of this valuable fish species [2,4,7,8].

SDDV is a double-stranded DNA virus belonging to the genus *Megalocytivirus*, family *Iridoviridae*. SDDV infections have been primarily observed in Asian seabass and reported in Southeast Asian countries including Thailand, Singapore, Malaysia, and Indonesia [1,3,6,7]. More recently, yellow seabream (*Acanthopagrus latus*) infected with SDDV were observed in China, exhibiting distinct clinical signs and pathological characteristics (swollen abdomen and ascites) compared to those observed in Asian seabass [9]. Understanding the genomic diversity of SDDV across Southeast Asia could potentially facilitate disease control and inform protective strategies against the disease. However, to date, only a few SDDV genome sequences have been deposited to public sequence databases [10,11]. The first partial genome sequence of SDDV was reported from Asian seabass in Singapore [4], followed by complete genomes from the same fish species in Thailand [12]. Additionally, an SDDV genome sequence was reported from yellow seabream in China [9]. A genome comparison between the first SDDV isolated in Singapore and the Thai SDDV revealed a high degree of sequence identity (99.97%), along with some notable variations, such as genome size and mutations [12]. Despite the regional significance of SDDV, there is limited information available regarding the genomic characteristics, strain diversity, and phylogenetic relationships of SDDV, particularly strains originating from different regions. In this context, genome characterization of different SDDV strains could elucidate factors influencing host susceptibility, virulence, and geographical distribution.

Metagenomics offers a powerful approach for characterizing viral and microbial communities, overcoming the limitations of traditional culturing methods [13]. This technique is particularly advantageous when prior knowledge of the organisms is scarce, co-infections are suspected, or comprehensive pathogen identification is required [14]. Several studies have successfully employed metagenomics to retrieve complete genomes of pathogens from diverse sample types [12,15,16].

This study aimed to use a metagenomic approach to investigate bacterial and viral communities associated with SDS in diseased Asian seabass isolated from a selection of Southeast Asian countries. We particularly focused on identifying potential pathogens co-infecting SDS-affected fish. Additionally, this study evaluated the potential of metagenomics for retrieving complete SDDV genomes and elucidating their phylogenetic relationships. By characterizing the complete genome of different SDDV strains, we aimed to gain a deeper understanding of the virus, which could ultimately inform the development of comprehensive disease prevention and control strategies for SDDV.

## 2. Materials and Methods

### 2.1. Fish Samples and DNA Extraction

A total of eight Asian seabass (*Lates calcarifer*) were collected from farms in Thailand, Singapore, and Malaysia during disease outbreaks from 2016 to 2019 from our previous studies (Table 1) [7,8]. A single fish collected from Singapore in 2019 was a juvenile (length 30 cm, weight 293 g), while the two fish collected from Malaysia in 2019 were adults (length 30–40 cm, weight 450–700 g). Fish from both countries were cultured in sea cages with water salinities ranging from 28–31 ppt. Fish collected from Thailand during 2016–2019 were juveniles (weights ranging from 60–280 g) cultured in floating cages with water salinity of 10 ppt. All fish displayed typical clinical signs of SDS including lethargy, darkened bodies, fin and tail rot, easily detached scales, severe scale loss, and hemorrhages, and the tissues collected included brain, kidney, liver, spleen, and fin. Tissues were preserved in RNAlater™ reagent (Thermo Fisher Scientific, Waltham, MA, USA) and kept at −20 °C until use. Genomic DNA was extracted using a conventional phenol/chloroform method [17]. All fish samples were screened for SDDV infection using SDDV-specific semi-nested conventional PCR as described by Charoenwai et al. [18] to confirm SDDV infection prior to sequencing.

### 2.2. Library Preparation and Sequencing

Genomic DNA extracted from fin (sample 7), kidney (sample 12), liver (sample 2, 3, 4, and 6), and pooled internal organs (kidney, spleen, and liver, sample 21 and 23) were subjected to metagenome sequencing. DNA concentrations of all samples were quantified using a Qubit^TM^ fluorometer (Thermo Scientific, USA) and adjusted to 100 ng/µL prior to sequencing. Sequence library preparation was carried out using NEBNext^®^ Ultra DNA Library Prep Kit for Illumina (NEB, Ipswich, MA, USA) following the manufacturer’s instructions. The libraries were sequenced using the Illumina HiSeq 1000 in 150 bp paired-end mode. The sample sequencing was carried out by Suzhou GENEWIZ Biotechnology company (Suzhou, China).

### 2.3. Sequence Analysis

#### 2.3.1. Reads Quality Control and Host Removal

After obtaining the metagenomic sequencing datasets, low-quality reads (Q < 20) and adapter sequences were removed using Trimmomatic v 0.39 [19]. Subsequently, host sequence reads were removed by mapping the reads of each of the samples to the Asian seabass reference genome (GCA_001640805.1) using Bowtie2 v 2.5.1 [20].

#### 2.3.2. Taxonomic Classification

The host-depleted reads were taxonomically classified according to the lowest common taxonomic ancestor (LCA) using Kraken2 v 2.1.3 with the NCBI viral and bacterial RefSeq complete genomes/proteins (downloaded August 2023) [21]. Virus and bacteria abundance estimates were reported at family level and normalized as a proportion of the sequencing reads in each of the samples using Bracken v 2.8 [22]. Moreover, the top 10 most abundant bacteria and virus families of each sample were determined in reads per million (RPM) using the formula: [the number of reads classified to a specific taxonomic group/total number of classified reads] × 10^6^. Relative taxa abundance bar plot and heatmap (based on RPM for each sample) were generated using ggplot2 [23] in R v 4.2.2 [24].

#### 2.3.3. Metagenome Assembly and SDDV Genome Recovery

The host-depleted reads were assembled using MEGAHIT v 1.2.9, and contigs shorter than 200 bp were discarded [25]. Sequence similarity searches of the assembled contigs were conducted using NCBI BLASTx v 2.13.0 [26] and the full NCBI non-redundant protein (nr) database (downloaded on August 2024) with e-value cut off 1 × 10^−5^ to identify viral contigs, specifically SDDV. Subsequently, the SDDV contigs were subtracted from the samples that contained relative abundance of SDDV more than 70% (sample 4, 6, 7, 12, 21, and 23). Draft SDDV genomes were further subjected to genome assembly quality assessment by mapping all reads obtained for each sample to the SDDV TH2019 genome (MN562489) using Bowtie2 v 2.5.1 to determine genome size, depth, and breadth of read coverages [20]. Additionally, genome completeness was evaluated using Benchmarking Universal Single-Copy Orthologs (BUSCO v 5.7.1) analysis based on the conserved gene set of the iridoviridae_odb10 database [27].

#### 2.3.4. SDDV Genome Annotation

Open reading frames (ORFs) were predicted using Prodigal v 2.6.3, and their functions were predicted based on NCBI BLASTp [26] searches against the NCBI nr and conserved domain (CD) database (downloaded in January 2023) [28]. Genome segments were reoriented using progressive MAUVE using the SDDV TH2019 genome (MN562489) as reference before comparative genome analysis [29]. For comparative genome analysis, all SDDV genomes available in NCBI, C4575 (NC_027778), and ZH-06/20 (OM037668), were included. Pairwise comparison of whole genome and multiple sequence alignment (MSA) was performed using MAFFT v 7 with the FFT-NS-I strategy [30]. Dot plots were generated using LAST local alignment implemented in MAFFT with SDDV TH2019 on the horizontal axis [30]. Nucleotide and amino acid sequence similarities of SDDV TH7_2019 against other SDDV genomes were estimated using BLAST v 2.13.0 [26] and visualized using pyGenomeViz v 0.4.4 [31].

#### 2.3.5. Comparative Genomics and Phylogenetic Analyses

Due to limitation of availability of gene sequences and completeness of the retrieved SDDV genomes in this study, maximum likelihood (ML) phylogenetic trees were constructed based on (i) whole genome sequences (WGS), (ii) single-nucleotide polymorphisms (SNPs), (iii) six concatenated iridoviruses core genes, and (iv) individual iridoviruses core genes. Sequences of megalocytiviruses (MCVs) including ISKNV (NC_003494), red seabream iridovirus (RSIV) (MK689686), European chub iridovirus (ECIV) (MK637631), and turbot reddish body iridovirus (TRBIV) (GQ273492) were included in the phylogenetic analysis while Singapore grouper iridovirus (SGIV) (AY521625) from family *Ranavirus* was used as an outgroup. A phylogenetic tree based on WGS was constructed using MAFFT-derived MSA and the IQ-TREE web interface accessed on 25 February 2024 with 1000 bootstraps through the ultrafast bootstrap approximation approach [32]. The best fit nucleotide substitution model was selected according to the lowest Bayesian information criterion (BIC) score using ModelFinder accessed on 25 February 2024 [33]. SNP calling with SDDV TH2019 as a reference and subsequent SNP-based phylogenetic analysis were carried out using the Call SNPs and Infer Phylogeny (CSI) web service (https://cge.food.dtu.dk/services/CSIPhylogeny/) accessed on 25 February 2024 [34]. All available iridoviruses core gene sequences including DNA polymerase, DNA-dependent RNA polymerase II alpha subunit (RPO), DNA-dependent RNA polymerase II beta subunit (RPO2), D5 family NTPase ATPase, NTPase, flap endonuclease, ATPase, myristoylated membrane protein, NIF-NLI interacting factor, Uvr/REP helicase, and major capsid protein (MCP) were retrieved from the SDDV genomes for phylogenetic analysis based on individual genes and concatenated core genes (Table S1). The six iridovirus core gene sequences (D5 family NTPase ATPase, flap endonuclease, ATPase, myristoylated membrane protein, NIF-NLI interacting factor, and Uvr/REP helicase) were concatenated and aligned using MEGA X v 10.2 [35], and the best fit nucleotide substitution models were selected according to the lowest BIC score. Maximum likelihood phylogenetic analyses were performed using MEGA X with 1000 bootstraps [35].

Nucleotide and amino acid substitutions within the core genes were determined manually using the previous sequence alignments, and SDDV TH2019 was used as reference for result interpretation. Tandem repeats were identified using Tandem Repeats Finder (TRF) with default parameters (https://tandem.bu.edu/trf/trf.html) accessed on 25 February 2024 [36].

Pan-genome analysis of the four complete SDDV genomes (TH7_2019, TH2019, C4575, and ZH-06/20) was carried out using Roary v 3.13.0 [37] with identity threshold of 95%. Genes present in three or more genomes were defined as core genes, whereas genes present in fewer than three genomes were defined as accessory genes.

## 3. Results

### 3.1. Taxonomic Profiles of Bacteria and Viruses

Metagenomic sequencing resulted in a total of 22 to 30 million reads per sample and contained approximately 87% to 94% of host reads. After read trimming and host removal, the number of sequences that remained for each sample ranged from 1.5 to 3.2 million. The host-depleted read pairs were subjected to taxonomic classification using Kraken2 and the percentage number of classified reads ranged from 1% to 6%, except sample 7, which exhibited a remarkable percentage of microbial reads (42.81%) compared with other samples (Table 2). Among these microbial reads, the percentages of reads representing bacteria ranged from 84% to 99% with sample 23 possessing the lowest and sample 7 the highest number of bacterial reads, respectively. The taxonomic analysis revealed a total of 479 bacterial families across all samples.

**Figure 1:**
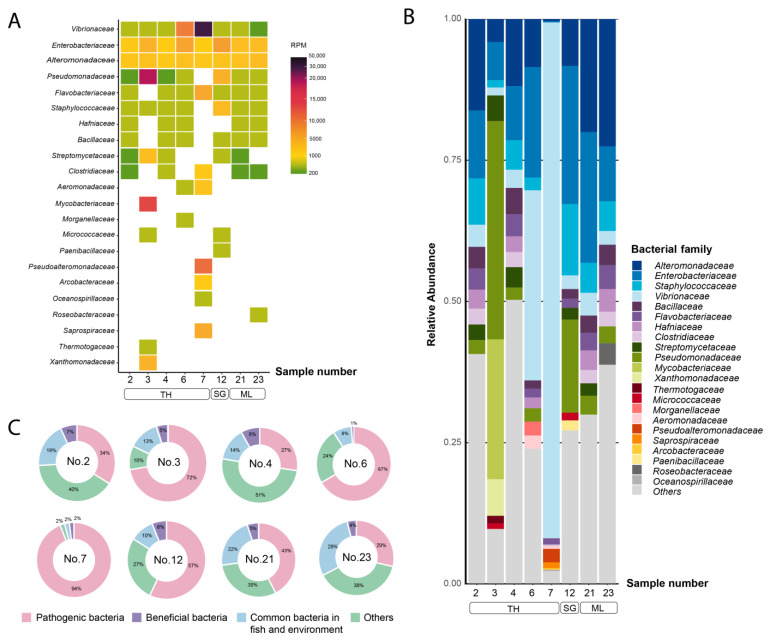
Taxonomic analysis of bacterial reads at family level. The top 10 bacterial families are shown. (**A**) Heat map represents reads per million (RPM) values. The RPM values are represented according to color gradient legend in the right panel. (**B**) Bar plot represents bacterial diversity in terms of relative abundance. (**C**) Pie charts represent proportion of bacterial families based on their impact on fish health. TH: Thailand, SG: Singapore, ML: Malaysia.

A heat map based on RPM was constructed to highlight the diversity of bacterial families across the samples (Figure 1A). The top 10 most abundant families present within each sample were selected for calculating family-level relative abundances, resulting in 22 dominant families (with the remaining families grouped as “others”) (Figure 1B). Among these, nine families were identified as containing species that are known fish pathogens, including *Staphylococcaceae*, *Enterobacteriaceae*, *Vibrionaceae*, *Flavobacteriaceae*, *Pseudomonadaceae*, *Morganellaceae*, *Hafniaceae*, *Mycobacteriaceae*, and *Aeromonadaceae*. They were represented by high proportions of the total bacterial reads obtained for several samples (Figure 1C). The top five most prevalent bacterial families were *Enterobacteriaceae*, *Alteromonadaceae*, *Vibrionaceae*, *Staphylococcaceae*, and *Flavobacteriaceae*. Notably, *Vibrionaceae*, Gram negative bacteria known for causing SDS, exhibited a remarkably high abundance in samples 6 (33%) and 7 (91%). Meanwhile, sample 3 displayed a distinct bacterial profile, characterized by a high abundance of *Mycobacteriaceae* (24%), which was absent in other samples, and a high abundance of *Pseudomonadaceae* (38%).

**Figure 2:**
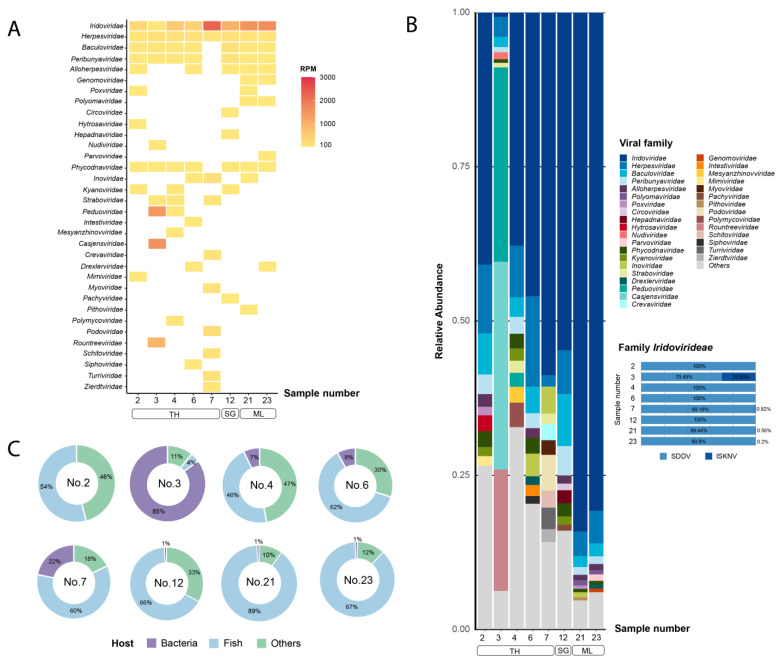
Taxonomic analysis of viral reads at family level. The top 10 viral families are shown. (**A**) Heat map represents reads per million (RPM) values. The RPM values are represented according to color gradient legend in the right panel. (**B**) Bar plot represents viral diversity in terms of relative abundance and proportion of species within the *Iridoviridae* family. (**C**) Pie charts represent proportion of viral families based on host. TH: Thailand, SG: Singapore, ML: Malaysia.

Regarding virus taxonomic analysis, a total of 146 virus families were identified. A heat map showing the abundance (RPM) and the diversity of the virus families among samples is shown in Figure 2A. The top 10 most abundant virus families in each sample were chosen for family-level relative abundances calculation, resulting in 34 dominant families (with the remaining families grouped as “others”) (Figure 2B). Among these, five families contain viruses that are known fish pathogens, while 22 families were representing bacteriophages. The top 5 most prevalent virus families were *Iridoviridae*, *Herpesviridae*, *Baculoviridae*, *Peribunyaviridae*, and *Phycodnaviridae*. The proportion of virus families known to be fish pathogens were dominated in all samples except sample 3, which exhibited a remarkably high proportion of bacteriophage families (85%) (Figure 2C). Notably, the family *Iridoviridae*, which includes SDDV, displayed the highest abundance in all samples except sample 3, which displayed its own unique viral community characteristics. Consistent with the bacterial taxonomic profile, sample 3 exhibited a unique virus profile with higher abundance of prokaryotic viruses, including *Casjensviridae* (33%), *Peduoviridae* (31%), and *Rountreeviridae* (19%). In all samples, *Iridoviridae* were predominantly represented by SDDV sequences, with the exception of sample 3 where approximately 30% of the *Iridoviridae* sequences were ISKNV sequences, while the remaining 70% were SDDV sequences (Figure 2B).

### 3.2. SDDV Genome Recovery

Following de novo assembly, the generated contigs were subjected to taxonomic classification. Notably, the largest contig (approximately 131 kb) was obtained from sample 7. SDDV contigs were successfully retrieved from seven out of eight samples, with the exception of sample 3. The SDDV genomes retrieved from samples 2, 4, 6, 7, 12, 21, and 23 were named as SDDV strain TH2_2016, TH4_2017, TH6_2018, TH7_2019, SG12_2019, ML21_2019, and ML23_2019, respectively. The assembled SDDV genomes exhibited varying degrees of coverage, ranging from 12% to 100% (sample 7; see Table 3). Furthermore, genome completeness was assessed using the BUSCO (Benchmarking Universal Single-Copy Orthologs) approach, evaluating a set of 10 single-copy iridovirus genes (Table S2). The result indicated the highest completeness within SDDV TH7_2019 genome with the following score: Complete (C): 10 (100%), Fragmented (F): 0, Missing (M): 0; followed by SDDV ML23_2019 with the following score: C: 8 (80%), F: 2 (20%), M: 0; and SDDV ML21_2023 with the following score: C: 7 (70%), F: 3 (30%), M: 0. Therefore, a complete genome was recovered only from sample 7 (SDDV TH7_2019) and submitted to NCBI under the accession no. PP660347. Apart from the BUSCO set of iridovirus genes, complete major capsid protein (MCP) gene sequences were obtained from SDDV ML23_2019 and submitted to NCBI (Table S2).

### 3.3. SDDV Genome Characterization

**Figure 3:**
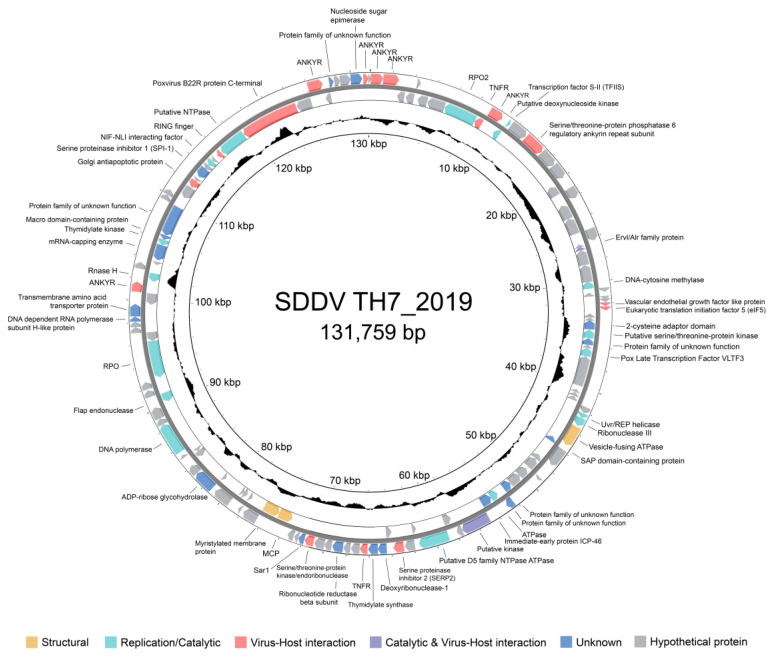
Circular genome map of the SDDV TH7_2019 genome. The outer and inner rings represent sense and antisense strands, respectively. Arrows indicate open reading frames (ORFs) and the direction of their transcripts. The ORFs are colored based on their functions.

The genome of SDDV strain TH7_2019 was subjected to genome annotation to predict ORFs and their potential function. The SDDV TH7_2019 genome size was 131,759 bp with a G + C content of 36.6% (Table 4). A total of 134 ORFs encoding putative proteins were predicted based on BLASTp searches against nr and CD database. A circular genome map of the SDDV TH7_2019 genome is shown in Figure 3. Detailed information on the predicted ORFs, including their lengths, homologous proteins based on BLASTp results, and predicted functions, is presented in Table S3. All predicted ORFs were homologous to SDDV and other iridovirus genes. Among these, 38 ORFs were functionally annotated, including 26 iridoviruses core genes. Functions of viral proteins were categorized into four types; (i) structural proteins: structural and virion-associated elements; (ii) replication/catalytic proteins: essential proteins in DNA replication and transcription, or support virus replication and adaptation, or cell signaling; (iii) virus–host interaction: virulence or immune evasion proteins; and (iv) unknown function. Several proteins that are crucial for the virus life cycle, including MCP (ORF079), DNA-dependent RNA polymerase II alpha (RPO) (ORF099) and beta (RPO2) (ORF008) subunit were identified. Moreover, viral proteins involved with virulence, including eukaryotic translational initiation factor 2α (eIF2α) (ORF032), ribonuclease III (ORF046), Erv1/Alr family (ORF023), and tumor necrosis factor receptor (TNFR) homologs (ORF009 and 069), were present.

**Figure 4:**
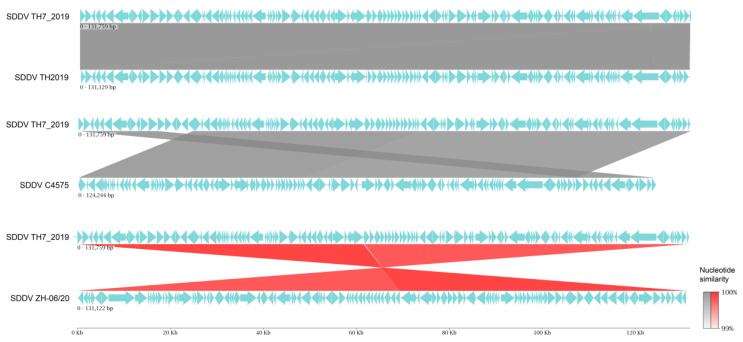
Linear map of whole genome sequence alignment of SDDV TH7_2019 against SDDV TH 2019, C4575, and ZH-06/20. Grey linkages indicate nucleotide similarity percentages, and red linkages indicate nucleotide similarity percentages of inverted sequences. The arrows indicate the direction of transcription.

Pairwise comparison demonstrated high similarity between the SDDV TH7_2019 genome and other previously published SDDV genomes. The SDDV TH7_2019 genome exhibited 99.80%, 99.71%, and 99.69% nucleotide identity with SDDV strains TH2019, ZH-06/20, and C4575, respectively (Figure 4). Dot plot analysis indicated substantial collinearity across the genomes, with the SDDV ZH-06/20 strain exhibiting an inverted direction (Figure S1). Gene-by-gene comparisons of predicted ORFs from the SDDV TH7_2019 genome indicated high amino acid similarity (<95% to 100%) for 110 to 122 ORFs of 134 ORFs when compared to other SDDV strains. Conversely, the number of ORFs exhibiting lower sequence similarity (<90%) were 6 to 14 (Table S4). Some of these variations can be attributed to the presence of varying numbers of tandem repeat sequences in ORFs. The most variable number of tandem repeats was found in ORF076 and ORF077 (SDDV TH7_2019), resulting in relatively low sequence similarities (78% to 79%) between Thai SDDV strains and other SDDV strains (Table S5). Variations in the number of tandem repeats were also observed in ORF051 and ORF088, resulting in sequence similarities of 97% and 98%, respectively.

### 3.4. Comparison Genomics and Phylogenetic Analysis

**Figure 5:**
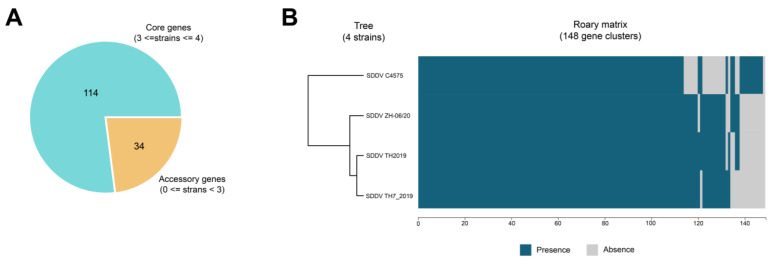
Pan-genome analysis of SDDV genomes. (**A**) Pie chart showing the number of core and accessory genes. (**B**) Matrix showing the distribution of core genes and accessory genes. Blue and grey indicate gene presence and absence, respectively. The tree represents the relationships based on gene presence/absence content. The x-axis shows the number of genes.

Pan-genome analysis identified a total of 148 putative protein-coding sequences among SDDV genomes including 114 core genes (present in at least three of four genomes) and 34 accessory genes (present in less than three genomes) (Figure 5). Predicted functions of the core genes are primarily involved with essential biological functions such as DNA replication, transcription, translation, and structural and cell metabolisms. Based on the presence and absence of genes, SDDV Thai strains (SDDV TH2019 and TH7_2019) were grouped into the same cluster whereas SDDV strains from Singapore (SDDV C4575) and China (SDDV ZH-06/20) were clustered separately. Compared to the other strains, SDDV C4575 possessed a unique pattern with ten and sixteen unique presence and absence genes, respectively. The functions of the unique accessory genes of SDDV C4575 are largely unknown, although some were predicted to encode for mRNA capping enzymes and signaling peptides. Similarly, the majority of accessory genes that were absent in SDDV C4575 were predicted to have unknown functions; however, some were predicted to contain signaling peptides and/or zinc ring finger motifs (see Supplemental Table S6).

Using SDDV TH2019 as reference, SNPs were identified across seven core genes (D5 family NTPase ATPase, flap endonuclease, ATPase, myristoylated membrane protein, NIF-NLI interacting factor, Uvr/REP helicase, and MCP) (Table S7). A total of 13 SNPs were identified and characterized, including five synonymous (not changing the amino acid sequence) and eight nonsynonymous (changing the amino acid sequence) variants. All nonsynonymous variants were missense variants. The SDDV ZH-06/20 exhibited the highest number of SNPs (7 SNPs), while none were detected in the SDDV TH7_2019. SDDV strains from Malaysia possessed unique SNPs within the NTPase, flap endonuclease, and NIF-NLI interacting factor genes. Additionally, variations of SNPs were found within the Uvr/REP helicase gene among the Thai strains.

**Figure 6:**
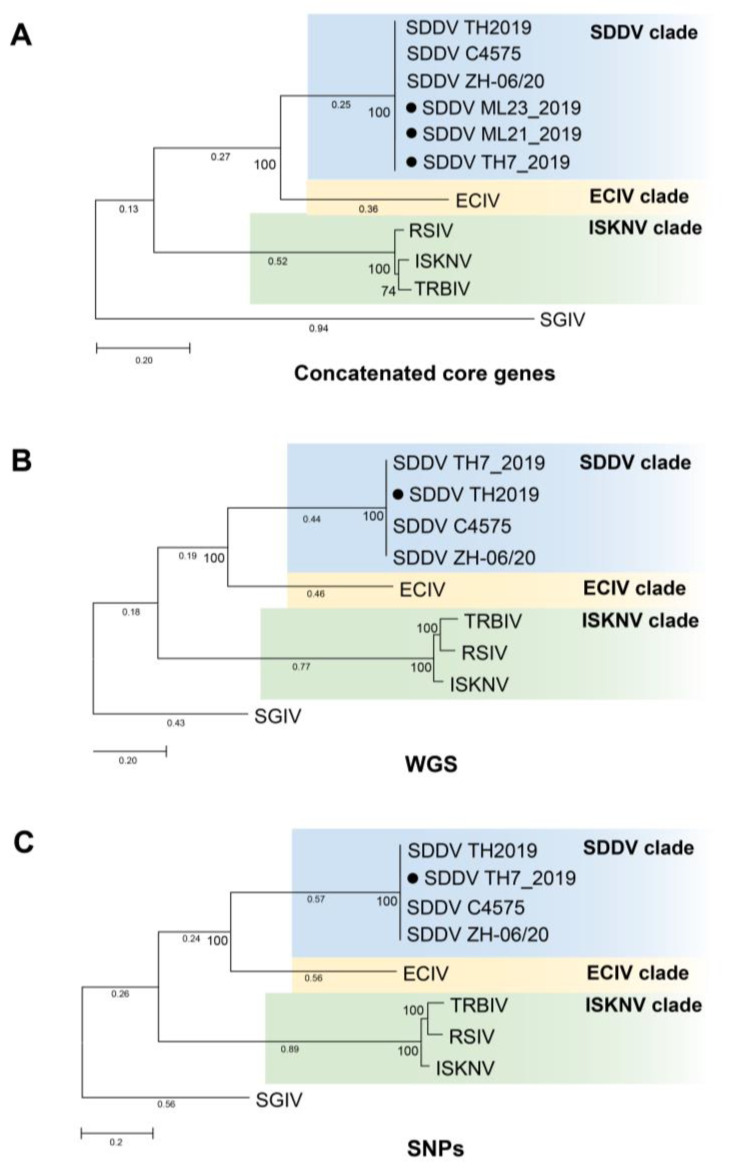
Maximum-likelihood tree based on (**A**) concatenated 6 core genes. The tree was constructed using MEGA X with General Time Reversible (GTR) + G nucleotide substitution model; (**B**) whole genome sequences (WGS). The tree was constructed using IQ TREE with GTR + F + G4 nucleotide substitution model; (**C**) single nucleotide polymorphisms (SNPs). The tree was constructed using IQ TREE with Transversion model (TVM) + F +ASC + G4 nucleotide substitution model. All trees were constructed with 1000 replications and bootstrap support values are shown at the nodes. Solid black circle represents SDDV strains from this study. Scale bar represents nucleotide substitution per site. Singapore grouper iridovirus (SGIV), belonging to the genus *Ranavirus*, was used as an outgroup. SDDV: scale drop disease virus, ECIV: European chub iridovirus, ISKNV: infectious spleen and kidney necrosis virus, RSIV: red sea bream iridovirus, TRBIV: turbot reddish body iridovirus.

**Figure 7:**
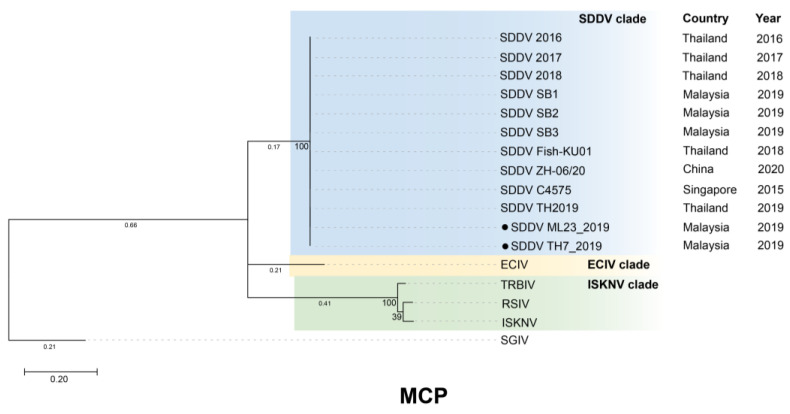
Maximum-likelihood tree based on major capsid protein (MCP) gene constructed using MEGA X software with Kimura 2-parameter (K2) + I nucleotide substitution model and 1000 replications. Scale bar represents nucleotide substitution per site. Solid black circle represents SDDV strains from this study. SGIV, belonging to the genus *Ranavirus*, was used as an outgroup. Bootstrap support values in percentage are shown at the tree node. SDDV: scale drop disease virus, ECIV: European chub iridovirus, ISKNV: infectious spleen and kidney necrosis virus, RSIV: red sea bream iridovirus, TRBIV: turbot reddish body iridovirus.

Phylogenetic analysis based on WGS, SNPs, and concatenated and individual core genes indicated that all SDDV strains clustered within a single clade, distinct from other iridoviruses (Figure 6, Figure 7, and Figures S2–S5). Notably, SDDV exhibited the closest relationships with ECIV followed by the ISKNV clade (ISKNV, RSIV, and TRBIV).

## 4. Discussion

Scale drop syndrome (SDS) has been a significant threat to Asian seabass aquaculture by causing severe economic losses across Southeast Asia [4,7,8]. Other pathogens, such as *V. harveyi* [5], *T. maritimum* [6], ISKNV [43], *L. calcarifer* herpes virus (LCHV) [44,45], and RSIV [46,47], can also cause similar symptoms or occasionally co-infected with SDDV. In this study, metagenomics analysis was used to study viral and bacterial communities within the tissues of diseased Asian seabass exhibiting pathological characteristics of SDS. Taxonomic analysis of bacteria identified members of the pathogenic bacterial families *Staphylococcaceae*, *Enterobacteriaceae*, *Vibrionaceae*, *Flavobacteriaceae*, *Pseudomonadaceae*, *Morganellaceae*, *Hafniaceae*, *Mycobacteriaceae*, and *Aeromonadaceae*. Among these, members of the *Vibrionaceae* family were suspected to be the cause of SDS observed in our samples, as its relative abundance was notably high. However, it is worth noting that only two samples exhibited a high abundance of the *Vibrionaceae* family. Most of the viral sequences in the samples represented members of the *Iridoviridae* family, predominantly SDDV. Based on the abundance of viral and bacterial reads, our findings suggest that SDDV and not *Vibrio* spp. was the primary pathogen responsible for causing SDS, while *Vibrio* spp. may also act opportunistically to increase disease severity.

In addition to these pathogens, sequences representing other potential pathogenic viruses and bacteria were discovered, raising concerns about opportunistic infections and disease transmission. For example, the *Herpesviridae* family was frequently identified as the second-ranked pathogen in many samples. This virus was reported to co-infect subclinically with SDDV in Asian seabass and may currently be endemic in this species [48]. For sample 3, which displayed the most distinctive bacterial and viral profiles, we observed a high abundance of the family *Pseudomonadaceae* and *Mycobacteriaceae*, corresponding with a high abundance of the bacteriophage family *Peduoviridae*. There is an increasing number of studies that demonstrated the role of the pathobiome in disease [49], and further work is needed to investigate the role of SDDV, *Vibrio* spp., and potentially other species in SDS.

Our study successfully employed metagenomics to recover a complete SDDV genome directly from the tissue samples, but this approach does have limitations. The detection of viral sequences can be challenging due to the overwhelming presence of host sequences and other microbial species. In our study, sample 7, which had a relatively high number of iridovirus reads, enabled de novo assembly of a single SDDV-like contig of 131 kb in length. This contig was confirmed as SDDV through reference genome mapping, comparative genomics, and phylogenetic analyses. However, samples with fewer iridovirus reads yielded only partial SDDV genomes. The completeness of viral genomes assembled via metagenomics depends on the abundance of viral reads in the samples, which could be influenced by the severity or stage of the viral infection. Therefore, a high sequencing depth is recommended for metagenomics analysis. Interestingly, sample 7 was obtained from fin tissue, which is generally considered to be a lower priority target organ for SDDV during the early stages of infection [48]. This aligns with a recent study by Charoenwai et al. [17], which also detected SDDV in non-destructive samples like mucus and fin clips.

The SDDV TH7_2019 genome was found to be 131,759 bp in length, with a GC content of 36.6%. This genome length is characteristic of MCV genomes, which are typically larger compared to other viruses in the *Iridoviridae* family. Recent research has categorized the *Megalocytivirus* genus into two main clusters, with one cluster, containing viruses like ISKNV, RSIV, and TRBIV, known as the ISKNV-like cluster. The other cluster is more distinct and includes SDDV and ECIV. This distinct grouping has led to the proposal of a new cluster named the SDDV-like cluster [9,50,51]. Our genome characterization and phylogenetic analysis support the proposal of an SDDV-like cluster, based on both GC content and genome length. The SDDV genome sequenced in this study, along with previously characterized SDDV and ECIV genomes, share a relatively low GC content (<40%, ranging from 36.5% to 37%) and a relatively larger size (>128 kb, ranging from 128 kbp to 131 kbp). These distinct features suggest that these viruses form a novel clade within the genus *Megalocytiviruses*, separate from other known MCVs [4,12,39,52]. Analysis of nucleotide and amino acid sequence similarities between SDDV genomes revealed a high degree of similarity (>99%) within the strains, even across different locations and years.

Interestingly, the first characterized SDDV genome (SDDV C4575) from Singapore has the shortest genome size (124 kb) compared to other SDDV genomes, which typically range around 131 kb [4,9,12]. A recent comparative genomics study comparing Thai SDDV strains TH2019 and SDDV C4575 revealed a 7.6 kb-long unique region encoding for ORFs 15–20 with unknown functions in SDDV TH2019 [12]. However, our study identified the presence of this region in SDDV SG12_2019, which originated from Singapore. The missing region in SDDV C4575 could potentially be either the variations within Singapore strains or a consequence of the limitations of the sequencing technique, VIDISCA-454 (virus discovery cDNA-AFLP combined with Roche 454). This method is often challenged by high interference from background sequences and limited availability of reference genomes, which may have resulted in incomplete sequencing of certain genomic regions [53,54]. Pan-genome analysis revealed a highly conserved genome structure shared by all SDDV strains with the majority of genes (70%) defined as SDDV core genes. It is a common occurrence for core genes to be primarily associated with genome replication, transcription, and modification, as they are widely recognized as essential genes across many viral species. Meanwhile, variations were observed among the accessory genes. SDDV C4575 exhibited the most distinct gene presence/absence pattern. Even though the functions of the majority of these genes are unknown, some are potentially linked to enhancing mRNA stability during the translation process. The absence of the 7.6 kb region, which contains six ORFs, in SDDV C4575 would have influenced the results of the pan-genome analysis. Further functional characterization of the accessory genes could elucidate potential differences contributing to virulence or adaptation ability among SDDV strains.

Gene prediction revealed 134 putative ORFs in the SDDV TH7_2019 genome, with known functions attributed to some genes. Out of these, 26 genes were identified as iridovirus core genes, showing high homology to SDDV and other viruses within the family. Notably, among these core genes, MCP (ORF079) is a structural component of the virus particles, constituting 40–50% of the viral particle [38]. The other iridovirus core genes are mostly associated with DNA replication, transcription, and cell metabolism. The genes described as essential for the viral life cycle are DNA polymerase (ORF008 and ORF099), DNA repair protein (ORF096), D5 family NTPase (ORF062), DNA binding/packing protein (ORF039), and helicase (ORF124) [38,55,56]. In addition to the essential genes, several genes associated with virulence and host immune interaction of SDDV were also identified. Some of these genes are related to host apoptosis manipulation, including tumor necrosis receptor (TNFR) homologs (ORF009 and ORF069) and Golgi antiapoptotic protein (ORF117). *Iridovirus*, like many other viral families including *Poxviridae*, possess genes that encode proteins capable of suppressing host apoptosis. Apoptosis is a natural cellular self-destruct mechanism that eliminates virus-infected cells. By inhibiting apoptosis, these viral genes prolong the survival of infected cells, creating a more favorable environment for viral replication [42]. ORF072 was identified as a gene encoding the small subunit of ribonucleotide reductase. This protein, previously studied in poxviruses, functions by binding to the host ribonucleotide reductase large subunit. This interaction induces host ribonucleotide reduction, thereby facilitating the viral replication process [57]. TNFR homologs or TNFR-associated protein genes are commonly found in other fish iridoviruses, whereas gene loss events have been reported in many non-fish iridoviruses, such as those affecting amphibians and reptiles [58]. These genes may have significantly contributed to the adaptation to different natural host species during iridovirus-host co-evolution [58]. Furthermore, the SDDV genome contains six ORFs encoding ankyrin repeat-containing proteins, which may encode repressors of the host immune response [51]. Collectively, the SDDV TH7_2019 genome consists of genes encoding host immune evasion functions. These genes potentially contribute to prolonged SDDV infection by inhibiting apoptosis and triggering inflammatory responses during infection. This aligns with observations of the delayed appearance of clinical signs of SDDV infection and host responses characterized by the release of chemokines, interleukins, and tumor necrosis factors [48].

Phylogenetic analyses utilizing various approaches, including WGS, SNPs, and core genes, consistently demonstrated a close relationship between SDDV strains, grouping them within a single clade. The high degree of conservation observed across the SDDV strains provides flexibility in the use of available resources for SDDV identification and classification. This observation supports the potential of utilizing the MCP and core genes, such as ATPase, which have been identified in several studies, for the development of diagnostic tools and vaccines [11,51,59,60].

Variations within SDDV strains were highlighted by determination of tandem repeats and SNPs. Tandem repeats are short lengths of DNA that are repeated multiple times, contributing to DNA slippage during the replication process. Variation in numbers of tandem repeats referred to as variable number tandem repeats (VNTRs) facilitates studies of genetic diversity and evolution. In SDDV, repeat sequences were previously reported in genes encoded for myristoylated membrane, hypothetical protein, ADP-ribose glycohydrolase, and putative ankyrin repeat protein through genome comparison between SDDV C4575 and TH2019 [12]. Similarly, our study identified the repeat sequences across these genes with additional SDDV genomes. Notably, SDDV strains from Thailand exhibited the same pattern and number of repeats, while SDDV strains from China and Singapore possessed similar number of repeats that were distinct from those found in Thai strains. A significantly different number of repeats were found in gene encoding putative membrane and hypothetical protein (ORF077 and ORF055) between Thai and other SDDV strains from Singapore and China. Several previous studies have demonstrated the potential of using repeat regions in epidemiological studies of viruses, such as white spot syndrome virus (WSSV) and African swine fever virus (ASFV), to trace the origin, investigate virus distribution and the development of genetic markers for strain discrimination [61,62,63,64]. Hence, these repeat sequences could potentially serve as genetic markers for further research on genetic diversity in SDDV.

Additionally, this study indicated variations in SNPs patterns across SDDV strains from different geographical origins by determining SNPs within the core genes. Notably, the SDDV strains from Malaysia in this study (SDDV ML21_2019 and SDDV ML23_2019) displayed unique missense mutations in genes encoding the NTPase, flap endonuclease, and NIF-NLI interacting factor. Missense mutations can alter the amino acid sequence, potentially affecting protein function. While the SDDV strains from China (SDDV ZH-06/20) harbored the highest number of SNPs, most were silent mutations, potentially having a less significant impact compared to the missense mutations observed in SDDV strains from Malaysia. Variations in SNPs were detected among Thai SDDV strains collected across different years in this study. Different SNPs were found at positions 29 (Gln29His) and 109 (Arg109Cys) of Uvr/REP helicase of SDDV TH6_2018 and TH4_2017, respectively, while no SNPs were found in SDDV TH7_2019. Previous reports have also highlighted differences in amino acid substitutions within genes encoding ATPase and myristoylated membrane proteins between SDDV isolates from different years [12]. This suggests a potential for temporal accumulation of mutations, leading to increased strain divergence over time. Furthermore, consistency was also observed across SDDV strains isolated from China, Malaysia, and Singapore, revealing the same SNPs within the DNA polymerase (Leu139Val and Arg590Arg) and RPO (Ala817Ala) genes. Viral mutations, particularly missense mutations, can be a mechanism for adaptation and survival under selective pressure [65]. Future studies should investigate whether these observed mutations impact the structural integrity or functionality of SDDV proteins, potentially influencing viral fitness or virulence, as observed in other viruses [66]. Nevertheless, the relatively small sample size and limited availability of SDDV genomes from diverse geographical regions of this study restrict the generalizability of our findings. Future research should prioritize expanding the SDDV strain collection by including more samples from a wider range of countries. This will provide a more comprehensive understanding of SDDV genetic diversity and potential geographical trends in mutation patterns.

Altogether, SDDV isolated from diseased Asian seabass from across Southeast Asian countries exhibited high similarity in their genomes and several genes still hold promise as targets for diagnostic approaches or vaccine development. However, differences between SDDV strains were also observed and these can likely be attributed to differences in both host and geographic location. This raises a concern related to the translocation of seabass stocks across countries, specifically the movement of fingerlings from hatcheries in one country to grow-out facilities in another. Such translocations could potentially introduce different viral strains into new environments, each with its own level of virulence and adaptability. As a result, the efficacy of existing diagnostic tools and vaccines in the affected countries could decrease over time. This highlights the need for ongoing updates in disease surveillance, as well as the implementation of effective control measures and virus prevention strategies.

## 5. Conclusions

This study employed metagenomic techniques to investigate the microbial diversity associated with scale drop disease (SDS) and successfully recovered a complete genome of the causative agent, scale drop disease virus (SDDV). We further characterized the SDDV genome through phylogenetic analysis and functional annotation, revealing insights into genome structure, evolutionary relationships, and potential virulence factors. These findings provide a foundation for developing targeted diagnostic tools and effective disease control strategies for SDDV.

## Figures and Tables

**Table 1:** The information of metagenomic samples collected from diseased fish.

Metagenomic Sample No.	Geographical Origin	Year	Organ
2	Chanthaburi, Thailand	2016	Liver
3	Chanthaburi, Thailand	2016	Liver
4	Chanthaburi, Thailand	2017	Liver
6	Chanthaburi, Thailand	2018	Liver
7	Chanthaburi, Thailand	2019	Fin
12	Singapore	2019	Kidney
21	Selangor, Malaysia	2019	Pool internal organs (liver, spleen, and kidney)
23	Selangor, Malaysia	2019	Pool internal organ (liver, spleen, and kidney)

**Table 2:** Classification of read counts of metagenomic samples using Kraken2.

Metagenomic Sample No.	Number of Raw Reads	Unclassified Reads (%)	Classified Reads (%)	Bacterial Reads (%)	Viral Reads (%)
2	1,577,409	98.90	1.10	93.13	6.87
3	3,012,060	94.16	5.84	92.04	7.96
4	3,144,439	98.70	1.30	88.75	11.25
6	1,422,958	97.30	2.70	96.62	3.38
7	2,844,173	57.19	42.81	99.09	0.91
12	2,017,399	97.55	2.45	92.54	7.46
21	1,515,485	98.77	1.23	85.10	14.90
23	2,096,503	98.69	1.31	84.42	15.58

**Table 3:** Assembly statistics of SDDV genomes retrieved from metagenomic samples.

Metagenomic Sample No.	Number of SDDV Contigs	SDDV Strain Name	Length (bp)	Percent Covered ^1^	BUSCO Search		
					Complete (C)	Fragmented (F)	Missing (M)
2	36	TH2_2016	16,035	12.3	1	0	9
4	126	TH4_2017	90,045	80.0	1	6	3
6	71	TH6_2018	30,800	23.1	1	2	7
7	1	TH7_2019	131,759	100	10	0	0
12	113	SG12_2019	108,163	81.9	3	7	0
21	79	ML21_2019	127,278	92.9	7	3	0
23	28	ML23_2019	130,742	98.9	8	2	0

^1^ Percent covered of query genomes compared with SDDV TH2019.

**Table 4:** General features of genomes used in this study.

Species	Isolate Name	Host	Year	Geographical Origin	GC (%)	Length (bp)	ORFs	GenBank Accession No.	Reference
Scale drop disease virus (SDDV)	TH7_2019	Asian seabass (*Lates calcarifer*)	2019	Thailand	36.6	131,759	134	PP660347	This study
Scale drop disease virus (SDDV)	TH2019	Asian seabass (*Lates calcarifer*)	2018	Thailand	36.6	131,192	135	MN562489	[12]
Scale drop disease virus (SDDV)	C4575	Asian seabass (*Lates calcarifer*)	2015	Singapore	37.0	124,244	129	KR139659	[4]
Scale drop disease virus (SDDV)	ZH-06/20	Yellow seabream (*Acanthopagrus latus*)	2020	China	36.56	131,122	135	OM037668	[9]
Infectious spleen and kidney necrosis virus (ISKNV)		Mandarin fish (*Siniperca chuatsi*)	2001	China	54.78	111,362	124	AF371960	[38]
European chub iridovirus (ECIV)	LEC15001	European chub (*Squalius cephalus*)	2005	United Kingdom	38.5	128,216	108	MK637631	[39]
Turbot reddish body iridovirus (TRBIV)		Turbot (*Scophthalmus maximus*)	2006	China	55.0	110,104	114	GQ273492	[40]
Red seabream iridovirus (RSIV)	KagYT-96	Japanese amberjack (*Seriola quinqueradiata*)	1996	Japan	53.0	112,719	117	MK689686	[41]
Singapore grouper iridovirus (SGIV)		Brown-spotted grouper (*Epinephelus tauvina*)	2004	Singapore	48.5	140,131	162	AY521625	[42]

## Data Availability

All sequence data presented in this study were submitted to the National Center for Biotechnology Information (NCBI) database. The original contributions presented in this study are included in the article/Supplementary Material, further inquiries can be directed to the corresponding authors.

## Supplementary Materials

The following supporting information can be downloaded at: https://www.mdpi.com/article/10.3390/ani14142097/s1, Figure S1: Dot plot of whole genome sequence alignment between the reference genome (SDDV TH 2019) and (A) C4575, (B) ZH-06/20, and (C) TH7_2019; Figure S2: Maximum-likelihood tree based on (A) DNA-dependent RNA polymerase II alpha subunit (RPO), (B) DNA-dependent RNA polymerase II beta subunit (RPO2), and (C) NTPase gene; Figure S3: Maximum-likelihood tree based on (A) ATPase, (B) myristoylated membrane protein, and (C) NIF-NLI interacting factor gene; Figure S4: Maximum-likelihood tree based on (A) D5 family NTPase ATPase and (B) DNA polymerase gene; Figure S5: Maximum-likelihood tree based on (A) Uvr/REP helicase and (B) flap endonuclease gene; Table S1: Information of gene sequences of SDDV used in this study; Table S2: BUSCO assessment results of recovered SDDV genomes in this study; Table S3: Genome annotation of the SDDV TH7_2019; Table S4: Gene-to-gene comparison of ORFs of the SDDV TH7_2019 against previously published SDDV genomes; Table S5: Tandem repeats in SDDV genomes; Table S6: Gene presence and absence of SDDV genomes; Table S7: Summary of single nucleotide polymorphisms (SNPs) in SDDV core genes.
