# Supplementary material for: Genome Characterization and Phylogenetic Analysis of Scale Drop Disease Virus Isolated from Asian Seabass (Lates calcarifer)
Source: Animals (Basel). 2024 Jul 18;14(14):2097. doi: 10.3390/ani14142097 (PMC11274154; doi:10.3390/ani14142097)
Supplement: Supplementary file 1 [file animals-14-02097-s001.zip › Revise_supplementary table.pdf]

Table S1. Information of gene sequences of SDDV used in this study

Isolate name	Host	Year	Geographical origin	Gene	Accession no.	Reference
TH4_2017	Asian seabass (*Lates calcarifer*)	2017	Thailand	Uvr/REP helicase	PP855146	This study
TH6_2018	Asian seabass (*Lates calcarifer*)	2018	Thailand	Uvr/REP helicase	PP855147	This study
SG12_2019	Asian seabass (*Lates calcarifer*)	2019	Singapore	Uvr/REP helicase	PP855148	This study
				NIF-NLI interacting factor	PP855151	This study
ML21_2019	Asian seabass (*Lates calcarifer*)	2019	Malaysia	D5 family NTPase ATPase	PP855160	This study
				Flap endonuclease	PP855155	This study
				ATPase	PP855162	This study
				Myristoylated membrane protein	PP855163	This study
				NIF-NLI interacting factor	PP855152	This study
				Uvr/REP helicase	PP855149	This study
ML23_2019	Asian seabass (*Lates calcarifer*)	2019	Malaysia	D5 family NTPase ATPase	PP855159	This study
				Flap endonuclease	PP855154	This study
				ATPase	PP855161	This study
				Myristoylated membrane protein	PP855164	This study
				NIF-NLI interacting factor	PP855153	This study
				Uvr/REP helicase	PP855150	This study
				DNA polymerase	PP855156	This study
				RPO	PP855157	This study
				MCP	PP889331	This study
SDDV Fish-KU01	Asian seabass (*Lates calcarifer*)	2018	Thailand	MCP	MN541862	[38]

SDDV 2016	Asian seabass (*Lates calcarifer*)	2016	Thailand	MCP	MH152403	[8]
SDDV 2017	Asian seabass (*Lates calcarifer*)	2017	Thailand	MCP	MH152404	[8]
SDDV 2018	Asian seabass (*Lates calcarifer*)	2018	Thailand	MCP	MH152405	[8]
SDDV SB1	Asian seabass (*Lates calcarifer*)	2019	Malaysia	MCP	MT012815	[7]
SDDV SB2	Asian seabass (*Lates calcarifer*)	2019	Malaysia	MCP	MT012816	[7]
SDDV SB3	Asian seabass (*Lates calcarifer*)	2019	Malaysia	MCP	MT012817	[7]

Table S2. BUSCO assessment results of recovered SDDV genomes in this study

BUSCO list	BUSCO search						
	SDDV TH2_2016	SDDV TH4_2017	SDDV TH6_2018	SDDV TH7_2019	SDDV SG12_2019	SDDV ML21_2019	SDDV ML23_2019
DNA polymerase	M	M	M	C	F	F	C
DNA-dependent RNA polymerase II alpha subunit (RPO)	M	F	M	C	F	F	C
DNA-dependent RNA polymerase II beta subunit (RPO2)	M	F	F	C	F	F	F
Putative D5 family NTPase ATPase	M	F	M	C	F	C	C
Putative NTPase	M	M	F	C	F	C	F
Flap endonuclease	M	F	M	C	C	C	C
ATPase	M	F	M	C	F	C	C
Myristoylated membrane protein	M	M	M	C	F	C	C
NIF-NLI interacting factor	C	F	M	C	C	C	C
Uvr/REP helicase	M	C	C	C	C	C	C
Non-BUSCO list	Blastp search						
MCP	n/a	n/a	n/a	1362 bp	n/a	516 bp	1362 bp

n/a: not applicable

Table S3. Genome annotation of the SDDV TH7_2019

ORF	Position (nt range)		Product size		Predicted function and conserved domain	Best BLAST hit		
	Start	end	nt (bp)	AA		Description	AA identity (%)	E-value
ORF001	31	1131	1101	377	Putative ankyrin repeat protein (ANKYR)	ORF115R [SDDV C4575]	100	0
ORF002	1147	2568	1422	474	Putative ankyrin repeat protein (ANKYR)	putative ankyrin repeat protein [SDDV TH2019]	100	0
ORF003	3259	2552	708	251	Hypothetical protein	ORF133 [SDDV ZH-06/20]	100	0
ORF004	4170	3385	786	262	Hypothetical protein	ORF118L [SDDV C4575]	100	0
ORF005	4197	4547	351	117	Hypothetical protein	ORF119R [SDDV C4575]	100	1e-83
ORF006	5482	4544	939	313	Hypothetical protein	ORF120L [SDDV C4575]	100	0
ORF007	7222	5528	1695	565	Hypothetical protein	ORF121L [SDDV C4575]	100	0
ORF008[a,b]	10409	7233	3177	1059	DNA-dependent RNA polymerase II beta subunit (RPO2)	ORF122L [SDDV C4575]	100	0
ORF009	11119	10406	714	238	Tumor necrosis factor receptor superfamily member 14 (TNFRSF14)	ORF123L [SDDV C4575]	100	0
ORF010	11188	12468	1281	427	Putative ankyrin repeat protein (ANKYR)	ORF124R [SDDV C4575]	100	0
ORF011[a]	13088	12465	624	208	Putative deoxynucleoside kinase	ORF125L [SDDV C4575]	99.52	9e-157
ORF012[a]	13099	13332	234	78	Transcription factor S-II (TFIIS)	SDDV C4575 ORF126R	100	7e-55
ORF013	13447	15096	1650	550	Hypothetical protein	hypothetical protein [SDDV TH2019]	100	0
ORF014	15144	17087	1944	648	Serine/threonine-protein phosphatase 6 regulatory ankyrin repeat subunit	Serine/threonine-protein phosphatase 6 regulatory ankyrin repeat subunit [SDDV TH2019]	100	0
ORF015	17103	18656	1554	518	Hypothetical protein	hypothetical protein [SDDV TH2019]	100	0
ORF016	18673	20049	1377	459	Hypothetical protein	hypothetical protein [SDDV TH2019]	100	0
ORF017	21062	20082	981	327	Hypothetical protein	hypothetical protein [SDDV TH2019]	100	0
ORF018	21140	22171	1032	344	Hypothetical protein	hypothetical protein [SDDV TH2019]	100	0

ORF019	23573	22161	1413	471	Hypothetical protein	hypothetical protein [SDDV TH2019]	100	0
ORF020	25157	23583	1575	525	Hypothetical protein	hypothetical protein [SDDV TH2019]	100	0
ORF021	25181	26281	1101	367	Hypothetical protein	ORF002R [SDDV C4575]	100	0
ORF022	26432	26298	135	45	Hypothetical protein	ORF114 [SDDV ZH-06/20]	100	1e-28
ORF023[a]	26811	26449	363	121	Ervl/Alr family protein	ORF003L [SDDV C4575]	100	4e-90
ORF024	28262	26817	1446	482	Hypothetical protein	ORF004L [SDDV C4575]	100	0
ORF025	29907	28264	1644	548	Hypothetical protein	ORF005L [SDDV C4575]	100	0
ORF026	30556	29861	696	232	DNA-cytosine methylase	Putative DNA (cytosine-5)-methyltransferase [SDDV TH2019]	100	3e-178
ORF027	30605	30988	384	128	Hypothetical protein	ORF008R	100	5e-95
ORF028	31306	30938	369	128	Hypothetical protein	ORF009L	100	3e-92
ORF029	31397	31582	186	62	Hypothetical protein	hypothetical protein [SDDV TH2019]	100	2e-40
ORF030	31563	31916	354	118	Hypothetical protein	ORF010R [SDDV C4575]	100	2e-84
ORF031	31929	32282	354	118	Vascular endothelial growth factor like protein	ORF011R [SDDV C4575]	100	7e-85
ORF032	32288	32665	378	126	C-terminal W2 domain of eukaryotic translation initiation factor 5 (eIF5)	ORF012R [SDDV C4575]	100	2e-88
ORF033	33307	32912	396	132	Hypothetical protein	ORF013L [SDDV C4575]	100	2e-97
ORF034	33555	33349	207	69	Hypothetical protein	hypothetical protein [SDDV TH2019]	100	9e-46
ORF035	34440	33562	879	293	2-cysteine adaptor domain	ORF014L [SDDV GF-MU1]	100	0
ORF036[a]	35289	34468	822	274	Putative serine/threonine-protein kinase	Putative serine/threonine-protein kinase [SDDV TH2019]	100	0
ORF037[a]	35927	35295	633	211	Protein family of unknown function (DUF5767)	ORF016L [SDDV C4575]	99.52	9e-157
ORF038	36230	35934	297	99	Hypothetical protein	hypothetical protein [SDDV TH2019]	100	3e-70
ORF039[a]	37048	36209	840	280	Poxvirus Late Transcription Factor VLTF3 like	ORF017L [SDDV C4575]	100	0
ORF040	39932	37053	2880	960	Hypothetical protein	ORF018L [SDDV C4575]	100	0
ORF041	40609	40322	288	127	Hypothetical protein	ORF019L [SDDV C4575]	100	3e-92
ORF042	41012	40641	372	124	Hypothetical protein	ORF021L [SDDV C4575]	100	9e-91
ORF043	41400	41020	381	127	Hypothetical protein	ORF022L [SDDV C4575]	100	2e-90

ORF044	41552	42112	561	187	Hypothetical protein	ORF023R [SDDV C4575]	100	1e-140
ORF045[a,b]	42149	42589	441	147	Uvr/REP helicase	ORF024R [SDDV C4575]	100	5e-109
ORF046[a]	42592	43365	774	258	Ribonuclease III	ORF025R [SDDV C4575]	100	0
ORF047	43485	45377	1893	631	Vesicle-fusing ATPase	Vesicle-fusing ATPase [SDDV TH2019]	99.84	0
ORF048	45834	45394	441	147	SAP domain-containing protein	ORF027L [SDDV C4575]	100	3e-106
ORF049	45860	47536	1677	559	Hypothetical protein	ORF028L [SDDV C4575]	100	0
ORF050	48045	47533	513	171	Hypothetical protein	hypothetical protein [SDDV TH2019]	100	2e-126
ORF051	49104	48058	1047	349	Hypothetical protein	ORF030 [SDDV GF-MU1]	100	0
ORF052	49132	49311	180	60	Hypothetical protein	hypothetical protein [SDDV TH2019]	100	3e-39
ORF053	50283	49333	951	317	Hypothetical protein	ORF031L [SDDV C4575]	100	0
ORF054	51416	50280	1137	379	Hypothetical protein	ORF032L [SDDV C4575]	100	0
ORF055[a]	52241	51432	810	270	Protein family of unknown function (DUF5832)	ORF033L [SDDV C4575]	100	0
ORF056	52274	52966	693	231	Protein family of unknown function (DUF2738)	ORF034R [SDDV C4575]	100	5e-175
ORF057[a,b]	53732	52995	738	246	ATPase	ORF035L [SDDV C4575]	100	0
ORF058[a]	54782	53739	1044	348	Immediate-early protein ICP-46 homolog	ORF036L [SDDV C4575]	100	0
ORF059[a]	54868	57483	2616	872	Putative kinase	ORF037R [SDDV C4575]	100	0
ORF060[a]	57494	58012	519	173	Hypothetical protein	ORF038R [SDDV C4575]	100	3e-127
ORF061[a]	58795	58055	741	247	Hypothetical protein	ORF039L [SDDV C4575]	99.59	0
ORF062[a,b]	58805	61537	2733	911	Putative D5 family NTPase ATPase (DNA primase)	putative helicase [SDDV TH2019]	100	0
ORF063	61893	61504	390	130	Hypothetical protein	SDDV C4575 (ORF042L)	99.22	2e-94
ORF064	61918	62826	909	303	Hypothetical protein	hypothetical protein [SDDV TH2019]	100	0
ORF065	62901	63848	948	316	Serine proteinase inhibitor 2 (SERP2)	SDDV C4575 (ORF045R)	100	0
ORF066	64415	63873	543	181	Hypothetical protein	SDDV C4575 (ORF046L)	100	4e-137
ORF067	64440	65246	807	269	Deoxyribonuclease-1	SDDV C4575 (ORF047R)	100	0
ORF068	65240	66103	864	288	Thymidylate synthase	thymidylate synthase [SDDV C4575]	100	0

ORF069	66151	66849	699	233	Tumor necrosis factor receptor superfamily member 1B (TNFR2)	ORF049R [SDDV C4575]	100	3e-172
ORF070	66861	67634	774	258	Hypothetical protein	hypothetical protein [SDDV TH2019]	100	0
ORF071	67681	68310	630	210	Hypothetical protein	ORF051R [SDDV C4575]	100	2e-158
ORF072[a]	68344	69309	966	322	Ribonucleotide reductase beta subunit	ORF052R [SDDV C4575]	100	0
ORF073	69375	70022	648	216	Hypothetical protein	ORF054R [SDDV C4575]	100	2e-161
ORF074	70027	70941	915	305	Hypothetical protein	hypothetical protein [SDDV TH2019]	100	0
ORF075	71010	71834	825	275	Serine/threonine-protein kinase/endoribonuclease	ORF056R [SDDV C4575]	100	0
ORF076	71863	72411	549	183	Sar1	ORF057R [SDDV C4575]	100	1e-137
ORF077	72422	72802	381	127	Hypothetical protein	hypothetical protein [SDDV TH2019]	100	8e-83
ORF078	72858	73424	567	189	Hypothetical protein	ORF059R [SDDV C4575]	100	1e-141
ORF079[a,b]	74878	73517	1362	454	Major capsid protein (MCP)	ORF060L [SDDV C4575]	100	0
ORF080[a,b]	76432	74882	1551	517	Myristoylated membrane protein	ORF061L [SDDV C4575]	100	0
ORF081	76494	77837	1344	488	Hypothetical protein	ORF062L [SDDV C4575]	100	0
ORF082	78080	77865	216	72	Hypothetical protein	hypothetical protein [SDDV TH2019]	100	2e-47
ORF083	78112	78381	270	90	Hypothetical protein	hypothetical protein [SDDV TH2019]	100	2e-63
ORF084	78765	78388	378	126	Hypothetical protein	ORF063L [SDDV C4575]	100	3e-93
ORF085	79029	78769	261	87	Hypothetical protein	hypothetical protein [SDDV TH2019]	98.84	9e-58
ORF086[a]	79333	80955	1623	541	Hypothetical protein	ORF065R [SDDV C4575]	100	0
ORF087	81273	80962	312	104	Hypothetical protein	ORF066L [SDDV C4575]	100	3e-77
ORF088	81310	83214	1905	635	ADP-ribose glycohydrolase	ADP-ribose glycohydrolase [SDDV TH2019]	100	0
ORF089	83220	84059	840	280	Hypothetical protein	hypothetical protein [SDDV TH2019]	99.64	0
ORF090	84699	84085	615	205	Hypothetical protein	hypothetical protein [SDDV TH2019]	100	2e-153
ORF091	85010	84714	297	99	Hypothetical protein	hypothetical protein [SDDV TH2019]	100	7e-69
ORF092	85044	85277	234	78	Hypothetical protein	hypothetical protein [SDDV TH2019]	100	1e-53
ORF093[a,b]	85622	88528	2907	969	DNA polymerase	DNA polymerase [SDDV TH2019]	100	0
ORF094	88531	89109	579	193	Hypothetical protein	hypothetical protein [SDDV TH2019]	100	6e-144
ORF095	89127	90200	1074	358	Hypothetical protein	hypothetical protein [SDDV TH2019]	100	0

ORF096[a,b]	91141	90224	918	306	Flap endonuclease	flap endonuclease [SDDV ZH-06/20]	100	0
ORF097	91225	91857	633	211	Hypothetical protein	ORF037 [SDDV ZH-06/20]	99.52	2e-158
ORF098	91946	92608	663	221	Hypothetical protein	ORF075R [SDDV C4575]	100	3e-162
ORF099[a,b]	96210	92659	3552	1184	DNA-dependent RNA polymerase II alpha subunit (RPO)	ORF076L [SDDV C4575]	100	0
ORF100	97063	96200	864	288	Hypothetical protein	ORF078L [SDDV C4575]	100	0
ORF101	97083	97601	519	173	Hypothetical protein	ORF079R [SDDV C4575]	100	2e-130
ORF102	97613	98110	498	166	Hypothetical protein	ORF080R [SDDV C4575]	100	3e-121
ORF103	98100	98564	465	155	DNA dependent RNA polymerase subunit H-like protein	ORF082R [SDDV C4575]	100	5e-116
ORF104	98571	99683	1113	371	Transmembrane amino acid transporter protein	ORF082R [SDDV C4575]	100	0
ORF105	100734	99652	1083	361	Hypothetical protein	ORF083L [SDDV C4575]	99.72	0
ORF106	100773	101687	915	305	Ankyrin repeat-containing protein	ORF084R [SDDV C4575]	100	0
ORF107	102677	101859	819	273	Rnase H	Rnase H [SDDV C4575]	100	0
ORF108	102861	103463	603	201	Hypothetical protein	ORF086R [SDDV C4575]	100	2e-150
ORF109	103804	103511	294	152	Hypothetical protein	ORF025 [SDDV ZH-06/20]	99.34	3e-111
ORF110	105469	103967	1503	501	mRNA-capping enzyme	mRNA-capping enzyme [SDDV TH2019]	100	0
ORF111	106035	105472	564	188	Thymidylate kinase	Thymidylate kinase [SDDV TH2019]	100	8e-142
ORF112	106470	106048	423	141	Macro domain-containing protein	ORF090L [SDDV C4575]	100	1e-105
ORF113[a]	109419	106495	2925	975	Protein family of unknown function (DUF5757)	Putative structural protein [SDDV TH2019]	100	0
ORF114	109447	109941	495	165	Hypothetical protein	ORF092R [SDDV C4575]	100	6e-120
ORF115	109949	110461	513	171	Hypothetical protein	ORF093R [SDDV C4575]	100	5e-126
ORF116	111670	110489	1182	394	Hypothetical protein	ORF094R [SDDV C4575]	100	0
ORF117	112442	111732	711	237	Golgi antiapoptotic protein (L6)	Protein lifeguard [SDDV TH2019]	100	1e-173
ORF118	112725	112480	246	82	Hypothetical protein	ORF096 [SDDV GF-MU1]	100	4e-57
ORF119	113940	112936	1005	335	Serine proteinase inhibitor 1 (SPI-1)	ORF097L [SDDV C4575]	100	0
ORF120	114370	113912	459	153	Hypothetical protein	ORF098L [SDDV C4575]	100	7e-113
ORF121[a,b]	114932	114375	558	186	NIF-NLI interacting factor	ORF099L [SDDV C4575]	100	2e-138

ORF122	115261	114938	324	108	Hypothetical protein	hypothetical protein [SDDV TH2019]	100	2e-78
ORF123	116087	115617	471	157	RING finger	ORF103L [SDDV C4575]	100	1e-107
ORF124[a,b]	118852	116141	2712	904	Putative NTPase	ORF104L [SDDV C4575]	100	0
ORF125	124541	118869	5673	1891	Poxvirus B22R protein C-terminal	ORF105L [SDDV C4575]	100	0
ORF126	126056	124593	1464	488	Hypothetical protein	ORF107L [SDDV C4575]	100	0
ORF127	126120	127463	1344	448	Ankyrin repeat-containing protein	ORF108R [SDDV C4575]	100	0
ORF128	127996	127448	549	183	Hypothetical protein	ORF109L [SDDV C4575]	100	2e-138
ORF129	128060	128467	408	136	Protein family of unknown function (DUF5850)	ORF110R [SDDV C4575]	100	1e-95
ORF130	128515	129063	549	183	Hypothetical protein	hypothetical protein [SDDV TH2019]	100	2e-135
ORF131[a]	129047	129991	945	315	Hypothetical protein	ORF112L [SDDV C4575]	100	0
ORF132	129995	131041	1047	349	Nucleoside sugar epimerase	nucleotide-sugar epimerase [SDDV C4575]	99.71	0
ORF133	131128	131595	468	156	Ankyrin repeat-containing protein	ORF114R [SDDV C4575]	100	1e-113
ORF134	131666	131758	92	31	Hypothetical protein	ORF115R [SDDV C4575]	100	3e-15

[a] iridovirus core genes, [b] gene defined by BUSCO

Table S4. Gene-to-gene comparison of ORFs of the SDDV TH7_2019 against previously published SDDV genomes. Data are shown in number ORFs within the ranges of amino acid similarity.

AA similarity (%)	Number of ORFs		
	SDDV TH_2019	SDDV ZH-06/20	SDDV C4575
100%	115	98	96
<100% - 95%	7	23	14
<95% - 90%	5	3	5
<90% - 85%	1	1	2
<85% - 80%	2	3	3
<80% - 75%	0	1	1
<75% - 70%	0	0	0
<70%	3	4	8

Table S5. Tandem repeats in SDDV genomes

Consensus pattern	Size (bp)	Location[a]	SDDV TH2019		SDDV TH7_2019		SDDV C4575		SDDV ZH-06/20	
			Percent match	Copy number	Percent match	Copy number	Percent match	Copy number	Percent match	Copy number
GATCTAGCAGGTACAGGTGGAGACAAGGAAG	31	ORF036	84	2.4	84	2.4	84	2.4	84	2.4
GGCGTAGGTTTG	12	ORF051	95	2.9	95	2.9	97	3.9	97	4.9
GATGATTCTGAGTGGGATACAATCAACGAAGAATCATCA	39	ORF064	92	2.3	92	2.3	92	2.3	92	2.3
TTTAAAACATTGATAATTG	19	ORF076-077	90	2.1	90	2.1	90	2.1	92	2.1
AACATCCGTGGCAAACGTTTGAAGGACAAG	30	ORF077	76	3.5	76	3.5	73	5.5	73	5.5
AACATCCGTGGCAAA	15	ORF077	75	3	75	3	65	7	65	7
TTGGCAAAAACATCCGTGGCAAACGTTTGAAGGACAAGAACATCCGTGGCAAAAACACAT	60	ORF077	100	1.9	100	1.9	100	2.9	100	2.9
ATGTCGTCGCATATGACACGGTAGCGGACGACGAAGCT	38	ORF088	88	7.1	88	7.1	87	6.1	86	6.1
TCGCATATGACACGGTAGCGGACGACGAAGCTATGTCAGTAGGCGTATGACACAGTAGCAGACGACGAAGCTATGTCGATCGCATACGACACAGTAGCGGACGACGAAGCTATCTCGA	118	ORF088	88	2.4	88	2.4	-	-	-	-
GGAGAAGCGCGTCGACGAGGAGACGCACGTCGT	33	non-coding region	89	2.2	89	2.2	89	2.2	89	2.2
ATGTTTGAACTACTA	15	ORF126	90	2.4	90	2.4	90	2.4	90	2.4
GGCGTAGGAGTCGGCGTAGGTTTG	24	ORF051	-	-	-	-	84	2.3	84	2.3
TATGACACGGTAGCAGACGACGAAGCTATGTCAGTCGCGTATGACACAGTAGCAGACGACGAAGCTATGTCGATCGCATACGACACAGTAGCGGACGACGAAGCTATGTCTGTTGCG	117	ORF088	-	-	-	-	92	2	92	2

[a] Location based on SDDV TH7_2019

Table S6 Gene presence and absence of SDDV genomes

Gene	Annotation	No. of isolates found	SDDV C4575	SDDV TH7_2019	SDDV TH2019	SDDV ZH-06/20
UNH60791.1	Serine/threonine-protein phosphatase 6 regulatory ankyrin repeat subunit	4	1	1	1	1
QLI60755.1	hypothetical protein	4	1	1	1	1
AKU37447.1	hypothetical protein	4	1	1	1	1
AKU37477.1	hypothetical protein	4	1	1	1	1
QLI60710.1	putative kinase	4	1	1	1	1
AKU37446.1	hypothetical protein	4	1	1	1	1
UNH60773.1	Eukaryotic translation initiation factor	4	1	1	1	1
UNH60701.1	hypothetical protein	4	1	1	1	1
AKU37534.1	hypothetical protein	4	1	1	1	1
UNH60721.1	hypothetical protein	4	1	1	1	1
UNH60758.1	putative ribonuclease	4	1	1	1	1
QLI60720.1	Tumor necrosis factor receptor superfamily member	4	1	1	1	1
AKU37449.1	hypothetical protein	4	1	1	1	1
QLI60654.1	hypothetical protein	4	1	1	1	1
QLI60671.1	hypothetical protein	4	1	1	1	1
AKU37460.1	Serine proteinase inhibitor	4	1	1	1	1
UNH60799.1	hypothetical protein	4	1	1	1	1
AKU37461.1	hypothetical protein	4	1	1	1	1
UNH60725.1	putative myristoylated protein	4	1	1	1	1
UNH60683.1	hypothetical protein	4	1	1	1	1
QLI60753.1	hypothetical protein	4	1	1	1	1
QLI60670.1	hypothetical protein	4	1	1	1	1
MCP	Major capsid protein	4	1	1	1	1
QLI60711.1	hypothetical protein	4	1	1	1	1
AKU37451.1	ORF_036L	4	1	1	1	1

UNH60704.1	DNA-directed RNA polymerase II subunit	4	1	1	1	1
UNH60754.1	hypothetical protein	4	1	1	1	1
UNH60734.1	hypothetical protein	4	1	1	1	1
AKU37430.1	putative serine/threonine-protein kinase	4	1	1	1	1
UNH60731.1	hypothetical protein	4	1	1	1	1
QLI60783.1	hypothetical protein	4	1	1	1	1
UNH60671.1	hypothetical protein	4	1	1	1	1
UNH60780.1	hypothetical protein	4	1	1	1	1
AKU37483.1	hypothetical protein	4	1	1	1	1
UNH60749.1	hypothetical protein	4	1	1	1	1
QLI60657.1	hypothetical protein	4	1	1	1	1
UNH60670.1	3 beta-hydroxysteroid dehydrogenase	4	1	1	1	1
QLI60737.1	hypothetical protein	4	1	1	1	1
UNH60709.1	hypothetical protein	4	1	1	1	1
AKU37542.1	hypothetical protein	4	1	1	1	1
UNH60741.1	hypothetical protein	4	1	1	1	1
AKU37487.1	hypothetical protein	4	1	1	1	1
AKU37454.1	hypothetical protein	4	1	1	1	1
QLI60678.1	hypothetical protein	4	1	1	1	1
AKU37455.1	putative helicase	4	1	1	1	1
AKU37485.1	DNA polymerase	4	1	1	1	1
UNH60760.1	hypothetical protein	4	1	1	1	1
AKU37498.1	hypothetical protein	4	1	1	1	1
QLI60686.1	hypothetical protein	4	1	1	1	1
AKU37471.1	Serine/threonine-protein kinase/endoribonuclease	4	1	1	1	1
RPO2	DNA-directed RNA polymerase II subunit	4	1	1	1	1
UNH60762.1	hypothetical protein	4	1	1	1	1
AKU37428.1	hypothetical protein	4	1	1	1	1
AKU37431.1	hypothetical protein	4	1	1	1	1
AKU37433.1	hypothetical protein	4	1	1	1	1
QLI60692.1	hypothetical protein	4	1	1	1	1

AKU37441.1	Vesicle-fusing ATPase	4	1	1	1	1
UNH60755.1	hypothetical protein	4	1	1	1	1
AKU37530.1	hypothetical protein	4	1	1	1	1
AKU37462.1	Deoxyribonuclease-1	4	1	1	1	1
AKU37463.1	Thymidylate synthase	4	1	1	1	1
UNH60735.1	hypothetical protein	4	1	1	1	1
QLI60738.1	hypothetical protein	4	1	1	1	1
QLI60739.1	ADP-ribose glycohydrolase	4	1	1	1	1
QLI60741.1	hypothetical protein	4	1	1	1	1
AKU37489.1	hypothetical protein	4	1	1	1	1
QLI60752.1	hypothetical protein	4	1	1	1	1
UNH60692.1	Thymidylate kinase	4	1	1	1	1
AKU37506.1	putative structural protein	4	1	1	1	1
AKU37421.1	putative DNA (cytosine-5)-methyltransferase	4	1	1	1	1
AKU37510.1	Protein lifeguard	4	1	1	1	1
UNH60685.1	mRNA decay activator protein	4	1	1	1	1
QLI60771.1	Serine proteinase inhibitor	4	1	1	1	1
QLI60774.1	hypothetical protein	4	1	1	1	1
UNH60679.1	putative RING finger protein	4	1	1	1	1
UNH60677.1	hypothetical protein	4	1	1	1	1
AKU37524.1	putative E3 ubiquitin-protein ligase makorin-1	4	1	1	1	1
AKU37525.1	hypothetical protein	4	1	1	1	1
AKU37531.1	putative ankyrin repeat protein	4	1	1	1	1
AKU37532.1	hypothetical protein	4	1	1	1	1
UNH60793.1	DNA-directed RNA polymerase III subunit	4	1	1	1	1
QLI60702.1	putative membrane protein	4	1	1	1	1
AKU37490.1	hypothetical protein	4	1	1	1	1
UNH60707.1	Flap endonuclease	4	1	1	1	1
QLI60766.1	hypothetical protein	4	1	1	1	1
AKU37418.1	putative FAD-linked sulfhydryl oxidase	4	1	1	1	1
QLI60779.1	hypothetical protein	4	1	1	1	1
AKU37439.1	hypothetical protein	4	1	1	1	1

AKU37508.1	hypothetical protein	4	1	1	1	1
UNH60732.1	hypothetical protein	4	1	1	1	1
AKU37539.1	putative ankyrin repeat protein	4	1	1	1	1
UNH60733.1	Ribonucleoside-diphosphate reductase subunit	4	1	1	1	1
AKU37503.1	mRNA-capping enzyme	4	1	1	1	1
UNH60775.1	hypothetical protein	4	1	1	1	1
UNH60756.1	putative SAP domain-containing protein	4	1	1	1	1
UNH60774.1	Vascular endothelial growth factor A	4	1	1	1	1
AKU37419.1	hypothetical protein	4	1	1	1	1
UNH60696.1	Ribonuclease	4	1	1	1	1
AKU37514.1	putative CTD phosphatase-like protein	4	1	1	1	1
UNH60747.1	hypothetical protein	4	1	1	1	1
AKU37432.1	putative transcription factor	4	1	1	1	1
QLI60768.1	hypothetical protein	4	1	1	1	1
AKU37505.1	ADP-ribose glycohydrolase	4	1	1	1	1
QLI60661.1	putative deoxynucleoside kinase	4	1	1	1	1
UNH60761.1	hypothetical protein	4	1	1	1	1
AKU37519.1	hypothetical protein	4	1	1	1	1
AKU37474.1	hypothetical protein	4	1	1	1	1
QLI60758.1	hypothetical protein	4	1	1	1	1
QLI60780.1	Serine/threonine-protein phosphatase 6 regulatory ankyrin repeat subunit B	4	1	1	1	1
AKU37501.1	hypothetical protein	4	1	1	1	1
AKU37538.1	Tumor necrosis factor receptor superfamily	4	1	1	1	1
UNH60699.1	Sodium-coupled neutral amino acid transporter	4	1	1	1	1
UNH60778.1	hypothetical protein	4	1	1	1	1
QLI60727.1	GTP-binding protein	4	1	1	1	1
QLI60666.1	hypothetical protein	3	0	1	1	1
UNH60787.1	hypothetical protein	3	0	1	1	1
QLI60672.1	hypothetical protein	3	0	1	1	1
UNH60720.1	hypothetical protein	3	0	1	1	1
UNH60712.1	hypothetical protein	3	0	1	1	1

UNH60694.1	hypothetical protein	3	0	1	1	1
QLI60714.1	hypothetical protein	3	1	1	1	0
QLI60775.1	Tripartite motif-containing protein	3	1	0	1	1
QLI60665.1	hypothetical protein	3	0	1	1	1
UNH60788.1	hypothetical protein	3	0	1	1	1
UNH60786.1	hypothetical protein	3	0	1	1	1
QLI60679.1	hypothetical protein	3	0	1	1	1
UNH60770.1	hypothetical protein	3	0	1	1	1
UNH60766.1	hypothetical protein	3	0	1	1	1
UNH60752.1	hypothetical protein	3	0	1	1	1
UNH60723.1	hypothetical protein	3	0	1	1	1
QLI60734.1	hypothetical protein	3	0	1	1	1
UNH60713.1	hypothetical protein	3	0	1	1	1
AKU37529.1	hypothetical protein	2	1	1	0	0
QLI60728.1	hypothetical protein	2	0	1	1	0
UNH60728.1	hypothetical protein	2	1	0	0	1
AKU37479.1	RING E3 ubiquitin ligase	2	1	0	0	1
UNH60772.1	hypothetical protein	2	0	0	1	1
UNH60711.1	hypothetical protein	2	0	0	1	1
AKU37544.1	ORF_129R	1	1	0	0	0
AKU37422.1	ORF_007L	1	1	0	0	0
AKU37435.1	ORF_020R	1	1	0	0	0
AKU37456.1	ORF_041L	1	1	0	0	0
AKU37459.1	ORF_044R	1	1	0	0	0
AKU37468.1	ORF_053L	1	1	0	0	0
AKU37492.1	ORF_077R	1	1	0	0	0
AKU37516.1	ORF_101R	1	1	0	0	0
AKU37521.1	ORF_106L	1	1	0	0	0
AKU37502.1	ORF_087L	1	1	0	0	0

Table S7. Summary of single nucleotide polymorphisms (SNPs) in SDDV core genes

	Nucleotide (NT) and amino acid (AA) substitutions															
	Thailand						Singapore				Malaysia				China	
	TH4_2017		TH6_2018		TH7_2019		C4575		SG12_2019		ML21_2019		ML23_2019		ZH-06/20	
Gene	NT	AA	NT	AA	NT	AA	NT	AA	NT	AA	NT	AA	NT	AA	NT	AA
DNA polymerase	n/a	n/a	n/a	n/a	-	-	415C>G	Leu139Val[a]	n/a	n/a	n/a	n/a	415C>G	Leu139Val[a]	415C>G	Leu139Val[a]
	n/a	n/a	n/a	n/a	-	-	1770G>A	Arg590Arg[b]	n/a	n/a	n/a	n/a	1770G>A	Arg590Arg[b]	1770G>A	Arg590Arg[b]
RPO	n/a	n/a	n/a	n/a	-	-	-	-	n/a	n/a	n/a	n/a	-	-	2433G>A	Glu811Glu[b]
	n/a	n/a	n/a	n/a	-	-	2451T>C	Ala817Ala[b]	n/a	n/a	n/a	n/a	2451T>C	Ala817Ala[b]	2451T>C	Ala817Ala[b]
RPO2	n/a	n/a	n/a	n/a	-	-	-	-	n/a	n/a	n/a	n/a	n/a	n/a	1395T>A	Ala465Ala[b]
D5 family NTPase ATPase	n/a	n/a	n/a	n/a	-	-	-	-	n/a	n/a	-	-	n/a	n/a	1243G>T	Ala415Ser[a]
NTPase	n/a	n/a	n/a	n/a	-	-	-	-	n/a	n/a	1990C>A	Pro664Thr[a]	n/a	n/a	-	-
	n/a	n/a	n/a	n/a	-	-	-	-	n/a	n/a	2703T>G	Ile901Met[a]	n/a	n/a	-	-
Flap endonuclease	n/a	n/a	n/a	n/a	-	-	-	-	n/a	n/a	404C>G	Pro135Arg[a]	404C>G	Pro135Arg[a]	-	-
ATPase	n/a	n/a	n/a	n/a	-	-	-	-	n/a	n/a	-	-	-	-	-	-
Myristoylated membrane protein	n/a	n/a	n/a	n/a	-	-	-	-	n/a	n/a	-	-	-	-	1368G>A	Pro456Pro[b]
NIF-NLI interacting factor	n/a	n/a	n/a	n/a	-	-	-	-	-	-	385A>C	Lys129Gln[a]	-	-	-	-
Uvr/REP helicase	-	-	87G>C	Gln29His[a]	-	-	-	-	-	-	-	-	-	-	-	-
	325C>T	Arg109Cys[a]	-	-	-	-	-	-	-	-	-	-	-	-	-	-

Dash represents no SNPs found. n/a: not applicable, [a] nonsynonymous variant, [b] synonymous variant
