# Supplementary material for: Genome Characterization and Phylogenetic Analysis of Scale Drop Disease Virus Isolated from Asian Seabass (Lates calcarifer)
Source: Animals (Basel). 2024 Jul 18;14(14):2097. doi: 10.3390/ani14142097 (PMC11274154; doi:10.3390/ani14142097)
Supplement: Supplementary file 1 [file animals-14-02097-s001.zip › Revise_supplementary_figure.pdf]

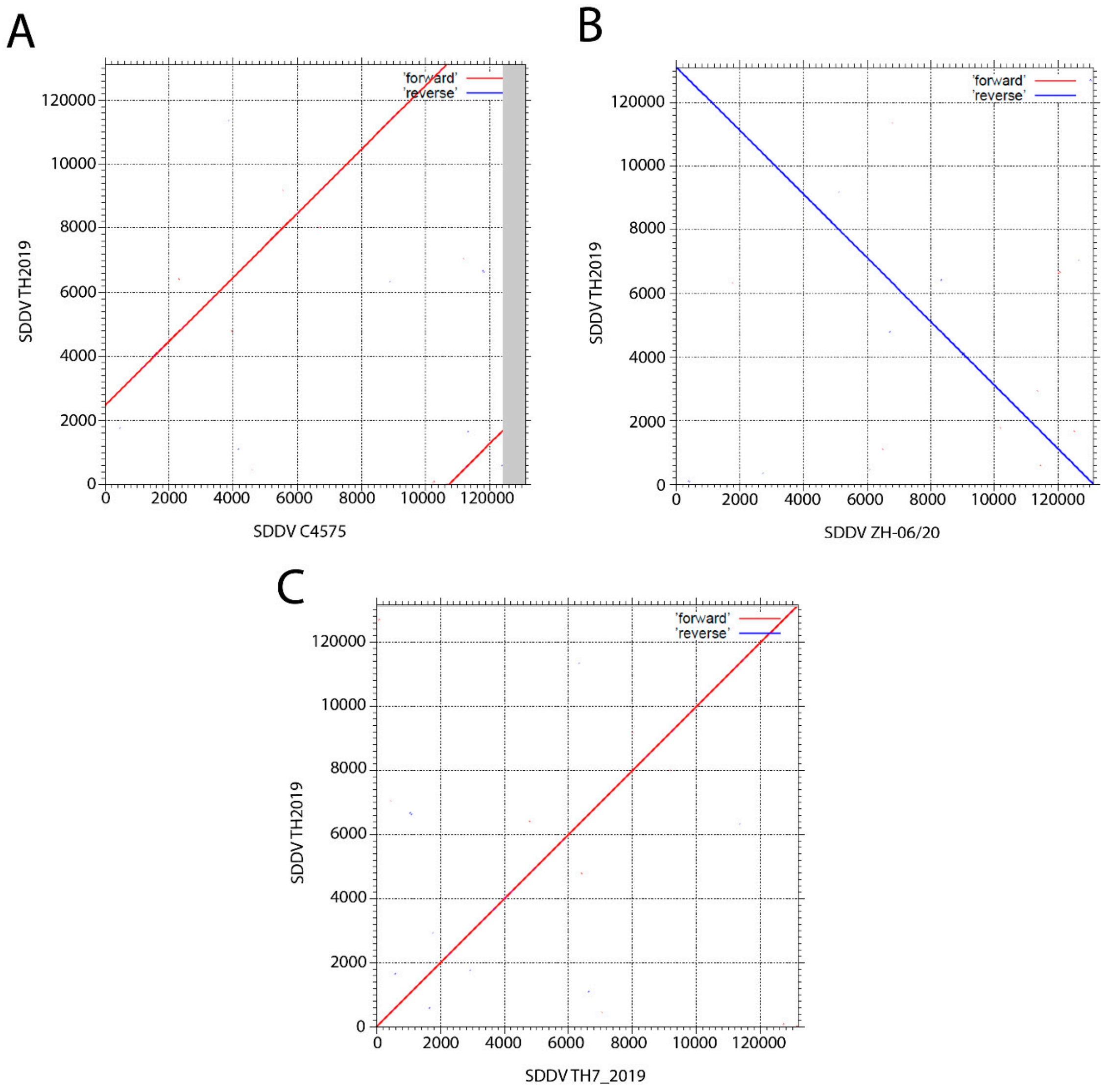

Figure S1. Dot plot of whole genome sequence alignment between the reference genome (SDDV TH2019) and SDDV (A) C4575, (B) ZH-06/20, and (C) TH7_2019.

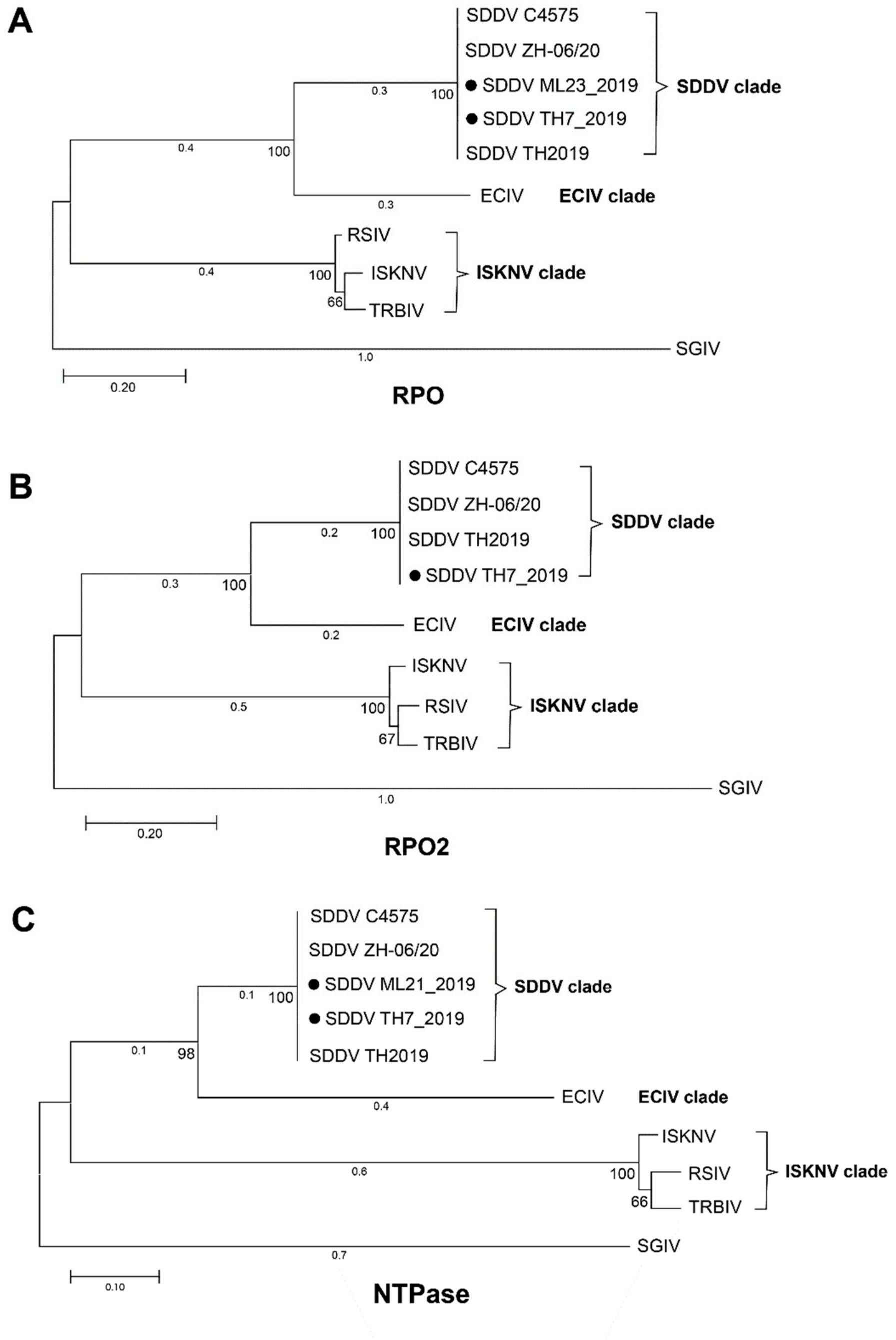

Figure S2. Maximum-likelihood tree based on (A) DNA-dependent RNA polymerase II alpha subunit (RPO), (B) DNA-dependent RNA polymerase II beta subunit (RPO2), and (C) NTPase gene constructed using MEGA X software with K2 + G nucleotide substitution model and 1,000 replications. Scale bar represents nucleotide substitution per site. Solid black circle represents SDDV strains from this study. SGIV, belonging to the genus *Ranavirus*, was used as an outgroup. Bootstrap support values in percentage are shown at the tree node.

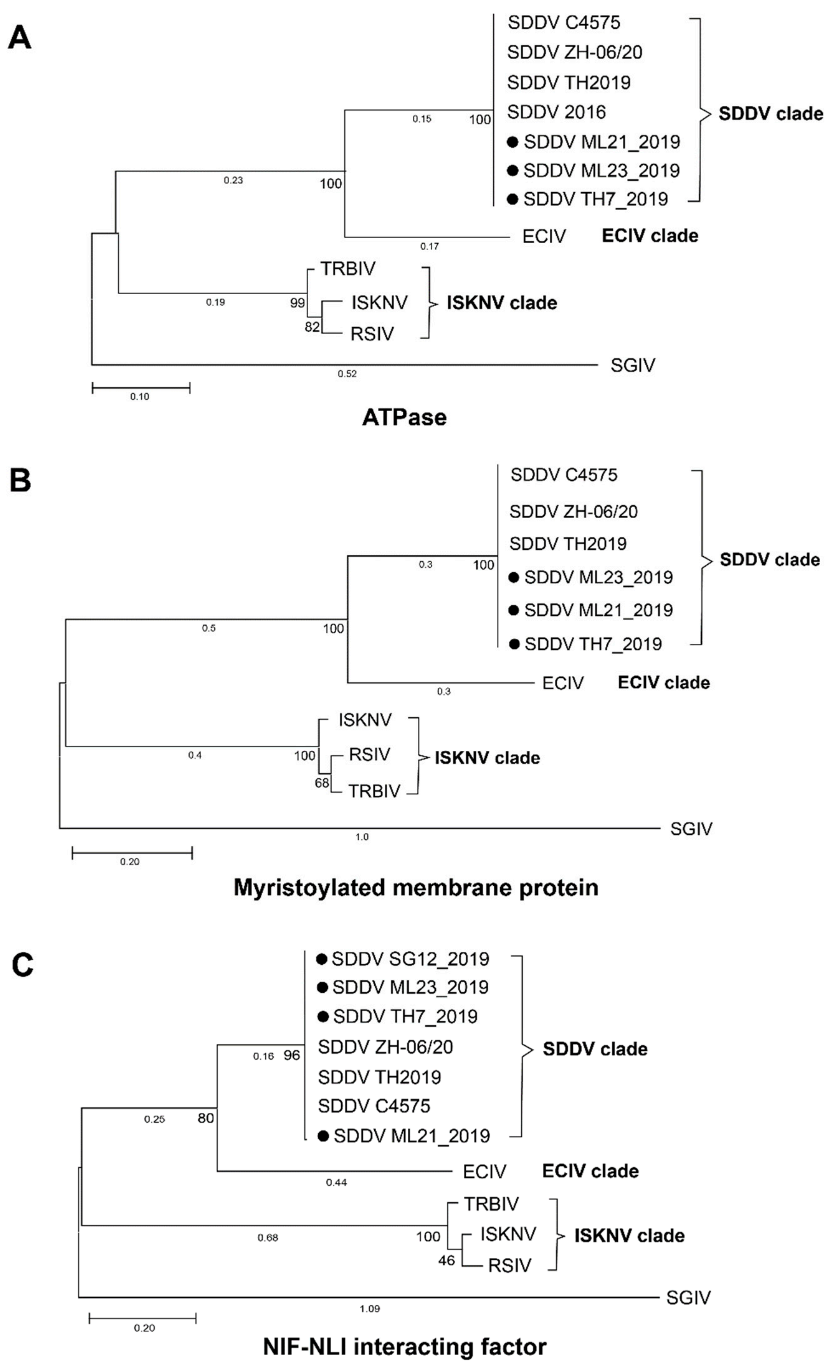

Figure S3. Maximum-likelihood tree based on (A) ATPase, (B) myristoylated membrane protein, and (C) NIF-NLI interacting factor gene constructed using MEGA X software with K2 + G nucleotide substitution model and 1,000 replications. Scale bar represents nucleotide substitution per site. Solid black circle represents SDDV strains from this study. SGIV, belonging to the genus *Ranavirus*, was used as an outgroup. Bootstrap support values in percentage are shown at the tree node.

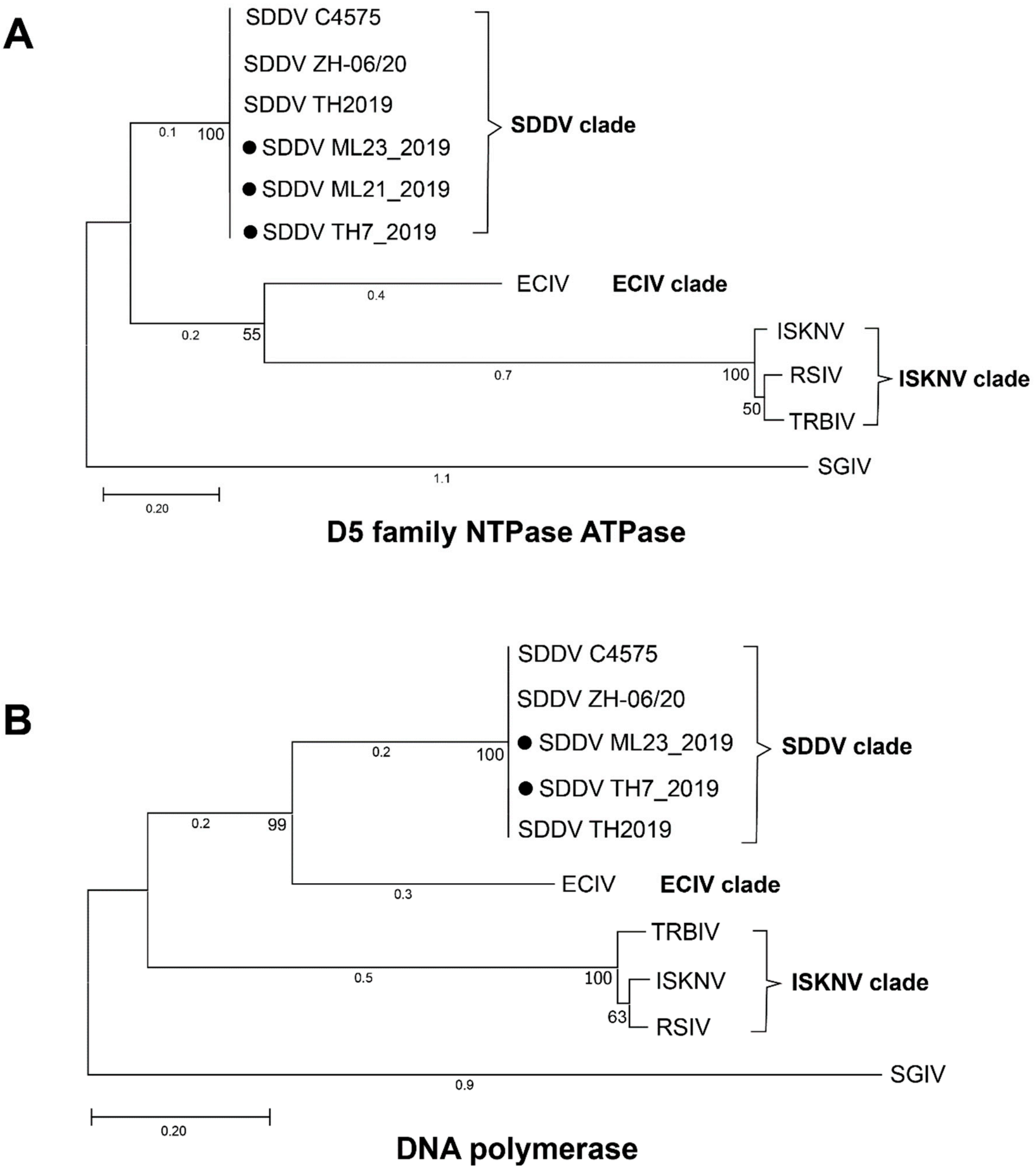

Figure S4. Maximum-likelihood tree based on (A) D5 family NTPase ATPase and (B) DNA polymerase gene constructed using MEGA X software with GTR + G nucleotide substitution model and 1,000 replications. Scale bar represents nucleotide substitution per site. Solid black circle represents SDDV strains from this study. SGIV, belonging to the genus *Ranavirus*, was used as an outgroup. Bootstrap support values in percentage are shown at the tree node.

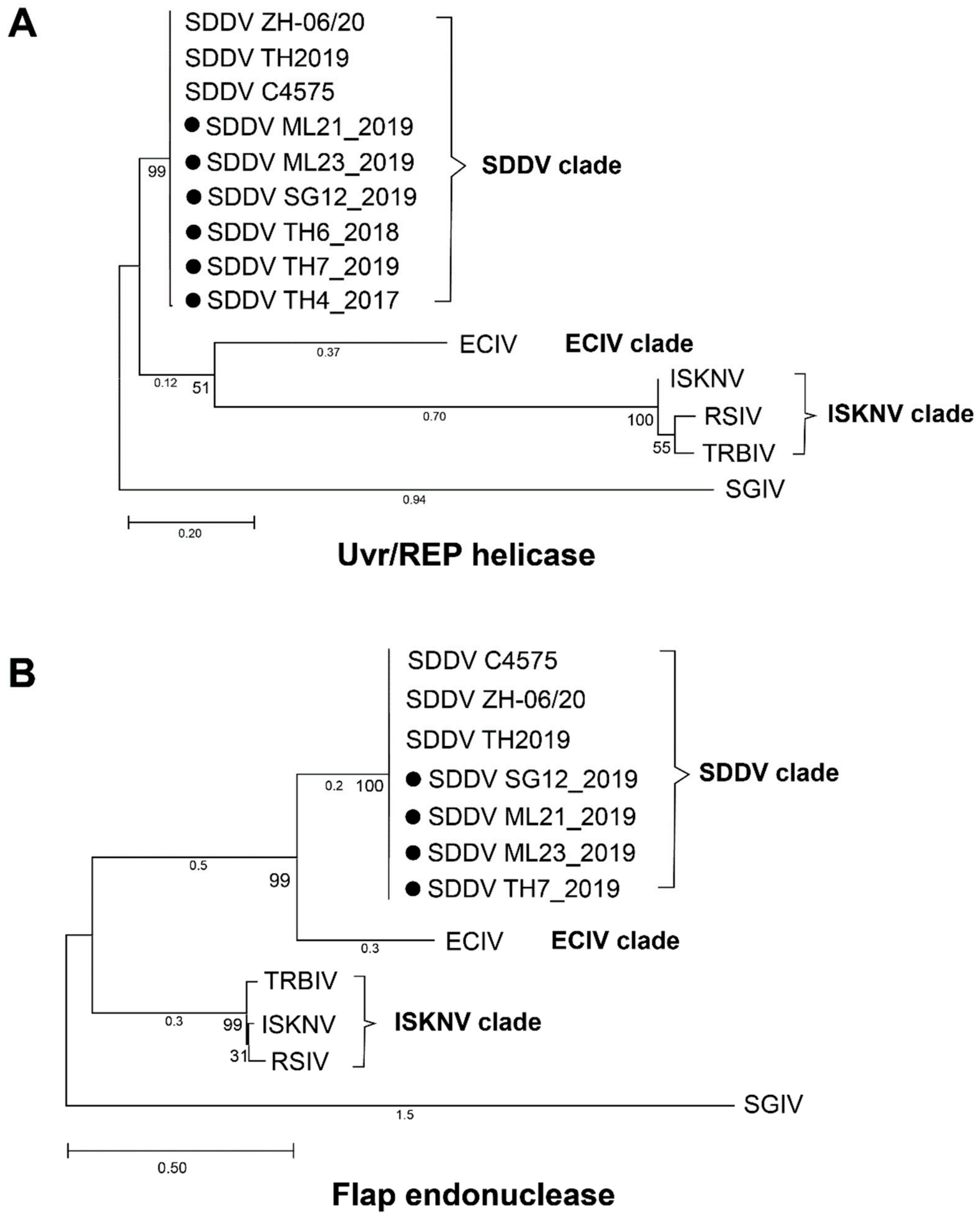

Figure S5. Maximum-likelihood tree based on (A) Uvr/REP helicase and (B) flap endonuclease gene constructed using MEGA X software with K2 + G + I nucleotide substitution model and 1,000 replications. Scale bar represents nucleotide substitution per site. Solid black circle represents SDDV strains from this study. SGIV, belonging to the genus *Ranavirus*, was used as an outgroup. Bootstrap support values in percentage are shown at the tree node.
